# Pinpointing genomic loci for drought-induced proline and hydrogen peroxide accumulation in bread wheat under field conditions

**DOI:** 10.1186/s12870-022-03943-9

**Published:** 2022-12-13

**Authors:** Mohammad Kamruzzaman, Mekides Abebe Beyene, Md Nurealam Siddiqui, Agim Ballvora, Jens Léon, Ali Ahmad Naz

**Affiliations:** 1 Institute of Crop Science and Resource Conservation (INRES)-Plant Breeding and Biotechnology, University of Bonn, Bonn, Germany; 2 Plant Breeding Division, Bangladesh Institute of Nuclear Agriculture (BINA), Mymensingh-2202, Bangladesh; 3 Department of Biochemistry and Molecular Biology, Bangabandhu Sheikh Mujibur Rahman Agricultural University, Gazipur 1706, Bangladesh; 4 Field Lab Campus Klein-Altendorf, University of Bonn, Bonn, Germany; 5 Department of Plant Breeding, University of Applied Sciences, Osnabrueck, Osnabrueck, Germany

**Keywords:** Drought, Proline, Hydrogen peroxide, GWAS, Wheat diversity

## Abstract

**Background:**

Proline (Pro) and hydrogen peroxide (H_2_O_2_) play a critical role in plants during drought adaptation. Genetic mapping for drought-induced Pro and H_2_O_2_ production under field conditions is very limited in crop plants since their phenotyping with large populations is labor-intensive. A genome-wide association study (GWAS) of a diversity panel comprised of 184 bread wheat cultivars grown in natural field (control) and rain-out shelter (drought) environments was performed to identify candidate loci and genes regulating Pro and H_2_O_2_ accumulation induced by drought.

**Results:**

The GWAS identified top significant marker-trait associations (MTAs) on 1A and 2A chromosomes, respectively for Pro and H_2_O_2_ in response to drought. Similarly, MTAs for stress tolerance index (STI) of Pro and H_2_O_2_ were identified on 5B and 1B chromosomes, respectively. Total 143 significant MTAs were identified including 36 and 71 were linked to drought and 2 and 34 were linked to STI for Pro and H_2_O_2,_ respectively. Next, linkage disequilibrium analysis revealed minor alleles of significant single-markers and haplotypes were associated with higher Pro and H_2_O_2_ accumulation under drought. Several putative candidate genes for Pro and H_2_O_2_ content encode proteins with kinase, transporter or protein-binding activities.

**Conclusions:**

The identified genetic factors associated with Pro and H_2_O_2_ biosynthesis underlying drought adaptation lay a fundamental basis for functional studies and future marker-assisted breeding programs.

**Supplementary Information:**

The online version contains supplementary material available at 10.1186/s12870-022-03943-9.

## Background

Water scarcity is a crucial aspect of crop production. When plants are subjected to water stress, they compensate by reducing shoot biomass resulting in yield losses of up to 70% [1, 2]. Water scarcity will become even more severe in the future, therefore, studies focusing on drought should be emphasized to develop drought-tolerant crop varieties. Plants deprived of water produce several biomolecules, including Proline (Pro) and Hydrogen peroxide (H_2_O_2_), which are important as they play multi-dimensional roles in drought. Pro is an amino acid that is synthesized in plants cells both under control and stress conditions [3]. At housekeeping levels under non-stress conditions, this amino acid is involved in normal plant growth. Whereas, dehydration events trigger an increase in its biosynthesis. Thus, Pro accumulation in plants follows a cyclic pattern under control-stress-control conditions. The common pathway for Pro biosynthesis in plants is the glutamic acid pathway. In this pathway, the amino acid glutamate is initially reduced to glutamic-γ-semialdehyde (GSA) by a pyrolline-5-carboxylate synthase. GSA is spontaneously converted to pyrroline-5-carboxylate (P5C), which is reduced to L-Pro by a P5C reductase [4]. In the catabolic reaction, a Pro dehydrogenase oxidizes L-Pro to P5C in the mitochondria and, finally, a P5C dehydrogenase converts P5C to L-glutamate [5, 6]. Pro biosynthesis varies between species under local environmental conditions and is regulated by different upstream and downstream signaling genes [7]. Drought conditions elicit up to a 100-fold increase in Pro content [8], which suggests that higher levels of Pro in different plant species could be associated with increased drought tolerance. Several efforts have been made to prove that higher Pro accumulation is associated with drought tolerance in different genotypes and plant species. But due to Pro having both enigmatic and distinct roles, these efforts met with limited success [9].

H_2_O_2_ is a common reactive oxygen species (ROS) in plants. Among ROS, H_2_O_2_ is relatively stable and measurable [10]. It is generated from the precursor O_2_^−^ mainly in mitochondria, chloroplasts and peroxisomes. As an important regulatory component in different signaling pathways, it is involved in many developmental and physiological processes in plants. At low concentrations H_2_O_2_ is beneficial and acts as a signaling molecule in physiological process such as photosynthesis, opening and closing of stomata, senescence, cell growth and development [11]. Its function under control conditions has been reported in *Arabidopsis*, maize, and Kentucky bluegrass [12, 13]. The raised amount of H_2_O_2_ observed under drought and other stress conditions results in a cross tolerance [14]. These tolerance mechanisms are modulated by the expression of resistance genes and antioxidant enzyme activities [15]. H_2_O_2_ overproduction may also trigger oxidative burst to organic molecules that causes programmed cell death [16]. Another report revealed that H_2_O_2_ is related to the stimulation of NADPH oxidation in plants under stress condition [17].

Physiological aspects and morphological traits are equally important since morphological attributes are directly linked to physiology and regulatory genes [18]. The susceptibility and tolerance of different cultivars are basically driven by contrasting physiology and gene function. While physiological characterization is prompt and precise, morphological characterization is often a lengthy process. Many studies have focused on morphological attributes, but the physiological status can be incorporated into a comprehensive approach to boost crop improvement programs [19]. Phenotyping physiological traits like Pro and H_2_O_2_ accumulation under field conditions is laborious, especially for a large number of accessions, but can help further the understanding of their role.

Many stress related genes are cumulatively involved with minor effects in drought tolerance mechanisms [20]. Moreover, the interaction between genes and the environment influence these mechanisms [20]. To understand the underlying regulatory elements, an appropriate genetic tool is required. Genome-wide association study (GWAS) is an approach that has been extensively used in plants to dissect complex traits both under normal and stress conditions [21, 22]. But studies towards identifying the key regulators of Pro and H_2_O_2_ accumulation are quite insufficient in plants and have yet to be performed in wheat. A previous GWAS of *Arabodopsis* accessions has shown a diversified response of root length to H_2_O_2_ exposure and identified a selective aquaporin gene which displayed the ability to channel H_2_O_2_ across cell membranes [23]. A global analysis of gene expression studies revealed that 1–2% of gene expression was regulated in response to H_2_O_2_ treatment under drought and other stress conditions [24]. A previous report performed GWAS and dissected the natural variation of Pro accumulation in *Arabidopsis* roots mediated by low water potential [25]. As Pro and H_2_O_2_ have multifunctional roles, they might be regulated by many genes [20], therefore, GWAS can be employed to uncover candidate loci and genes. With about 18 Gbp, the wheat genome is approximately 136 times larger than the *Arabidopsis* genome and also larger than that of other important crops like rice and maize. Therefore, wheat has more genetic potential and might reveal new genetic components for Pro and H_2_O_2_ metabolism. Considering this background, the present study aimed to [1] assess the diversity of Pro and H_2_O_2_ content under control and drought conditions, [2] determine the correlation of Pro and H_2_O_2_ with yield attributes and [3] identify loci for drought induced Pro and H_2_O_2_ accumulation under field condition.

## Results

### Diversity panel showed significant phenotypic variation for drought-induced pro and H_2_O_2_ accumulation

In order to observe phenotypic diversity induced by drought, we estimated and analyzed Pro and H_2_O_2_ content both under control and drought conditions. We observed a significant variation in accumulation of Pro and H_2_O_2_ among the cultivars under both control and drought conditions. Pro accumulated under control conditions to a minimum of 27.92 μg/g fresh weight (FW) and a maximum of 165.40 μg/g FW with a mean value of 83.72 μg/g FW (Table 1). Under drought conditions, a similar minimum of 84.51 μg/g FW, but a much higher maximum of 2420.55 μg/g FW were recorded, resulting in a mean of 929.49 μg/g FW. A Similar accumulation was observed in case of H_2_O_2_ (Table 1). The coefficient of variation was higher under drought conditions than under control conditions for both Pro and H_2_O_2_. Interestingly, the average Pro content of plants grown under drought condition was observed to be 11.10 times higher than under control conditions. In contrast, H_2_O_2_ content under drought was only 0.63 times higher compared to the controls. Analysis of variance results showed that genotype and treatment interactions were highly significant (*P* < 0.001) (Table 1). Under drought conditions, Zobel was the cultivar producing the lowest Pro amount, whereas the highest was observed in the variety Kurt (Supplementary Table S2). For H_2_O_2,_ Urban and Elixer respectively accumulated the lowest and highest amount under drought conditions (Supplementary Table S2). Both the highest and lowest Pro and H_2_O_2_ producing cultivars originated from Germany.

**Table 1:** Descriptive statistics and analysis of variance (ANOVA) based on average phenotypic values (μg/g fresh weight) of Pro and H_2_O_2_ under control, drought stress conditions

Traits	Max	Min	Mean	CV (%)	*C	Two-way ANOVA		
						G	T	G × T
Pro_con	165.40	27.92	83.72	11.97		***	***	***
Pro_dro	2420.55	84.51	929.49	27.16	11.10			
Pro_STI	77.54	0.66	13.85	–				
H_2_O_2__con	133.05	46.20	84.50	10.58		***	***	***
H_2_O_2__dro	216.53	78.09	132.52	13.86	0.63			
H_2_O_2__STI	3.82	0.73	1.56	–				

Abbreviations: *Pro_con,* proline content under control conditions; *Pro_dro,* Proline content under drought conditions; *H*_*2*_*O*_*2_*_*con,* H_2_O_2_ content under control conditions, *H*_*2*_*O*_*2_*_*dro,* H_2_O_2_ content under drought conditions *G,* Genotype; *T,* Treatment; *C, multiplied by change of phenotypic value under drought stress in comparison to the control

### Phenotypic observation of drought-induced pro and H_2_O_2_ accumulation for cultivar-origin and modern-traditional categories

**Fig. 1:**
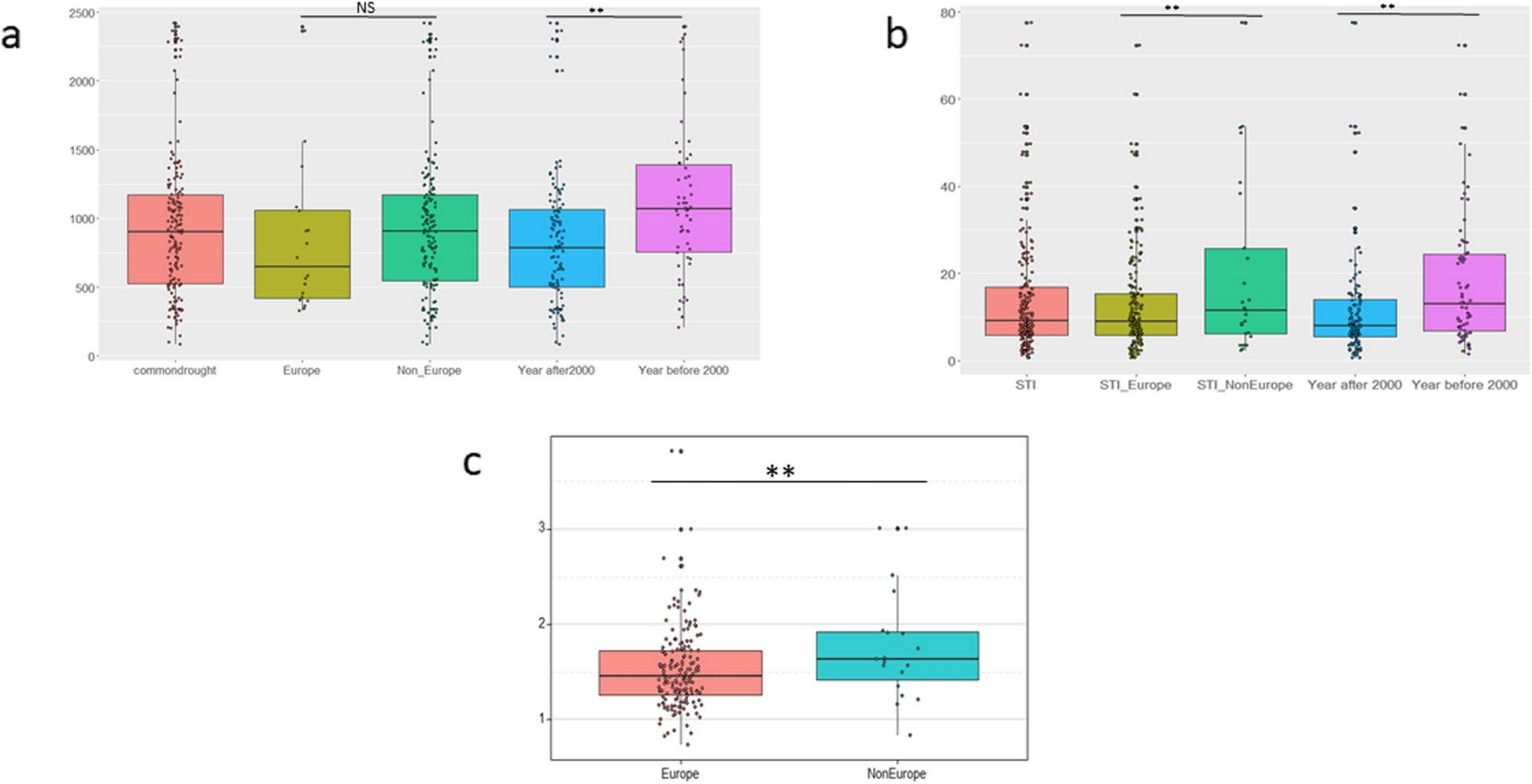
Pro accumulation of 184 the cultivars among different sub-groups. Pro accumulation of European and Non-European sub-groups and cultivars sub-groups registered before (traditional) and after the year 2000 (modern) under drought stress treatment in the field **a**; STI of Pro between STI of Europe (STI for Europe) and STI of Non-Europe (STI for Non-Europe) and between the cultivar group modern and traditional **b**; STI value of hydrogen peroxide between Europe and Non-European group **c**. **, Significant (*P* < 0.05); NS, Non-significant

The population genetic study on this panel was performed in a previous study and identified three population sub-groups [26]. Based on this, we further observed the effect of sub-group on phenotype. We analyzed Pro content under drought conditions and determined the STI of both Pro and H_2_O_2_ according to the origin of cultivars (Europe and non-Europe) and the year of their release before (traditional) or after (modern) the year 2000. A student’s t-test was performed to compare the sub-groups. No significant Pro difference was observed between the Europe and non-Europe sub-groups under drought stress (Fig. 1a). But the modern cultivars had significantly lower (*P* < 0.05) Pro contents than the traditional ones. In case of the STI of Pro, significant differences were found between both the Europe and non-Europe and the modern and traditional sub-groups (Fig. 1b). We performed similar analyses for the STI of H_2_O_2_. The analysis found a contrasting phenotypic difference between the Europe and non-Europe cultivar groups (Fig. 1c).

### Correlation analyses revealed a weak correlation of pro and H_2_O_2_ with yield attributes under drought condition

To know whether Pro and H_2_O_2_ are linked with yield attributes, we studied the correlation of yield-related attributes and Pro or H_2_O_2_ accumulation under control and drought conditions. Variable correlations were found. We observed that Pro has both positive and negative correlations with yield parameters under drought conditions (Supplementary Table S7a). The highest positive correlation was observed between Pro and plant height (PH). In contrast, the highest negative correlation was identified between Pro and grain yield (GY). H_2_O_2_ content under drought conditions correlated positively with the spike number (SN) and negatively with the thousand kernel weight (TKW) (Supplementary Table S7a). Under control conditions, Pro and H_2_O_2_ showed positive correlations with PH, GY and TKW (Supplementary Table S7b). In general, our findings revealed that correlations between Pro, H_2_O_2_ and yield attributes under control and drought treatments were not strong.

### GWAS identified candidate loci for drought- induced pro and H_2_O_2_ accumulation

**Fig. 2:**
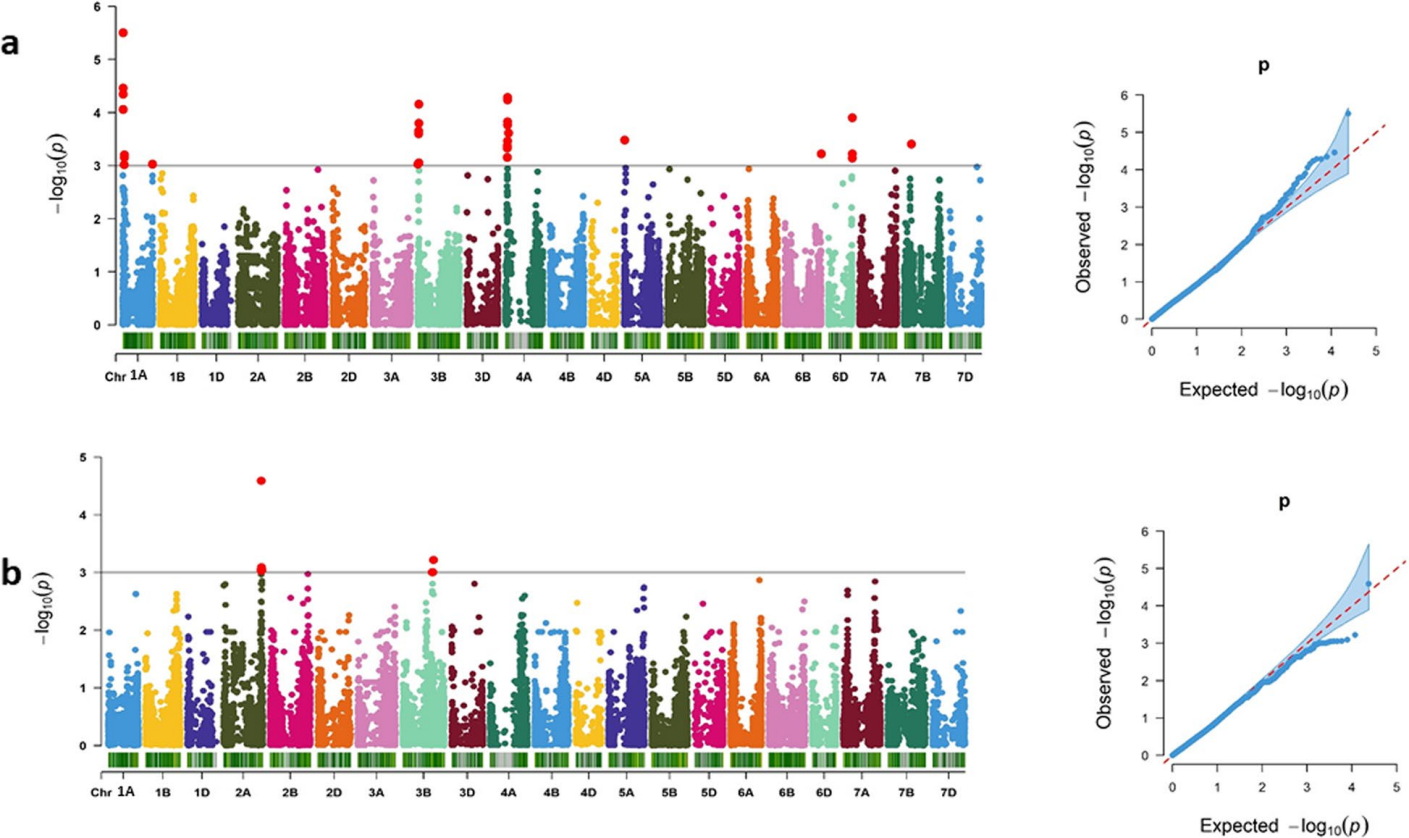
Manhattan and Q-Q plots of the GWAS on Pro and H_2_O_2_ content under drought conditions. Manhattan plot (left) and Q-Q plot (right) of Pro content under drought conditions **a** and Manhattan plot (left) and Q-Q plot (right) of H_2_O_2_ content under drought conditions **b**. − log_10_ (*P*) = 3.0 is the significant threshold level for MTA and marked by a horizontal grey line in the Manhattan plots. Red dots designate significant markers

**Fig. 3:**
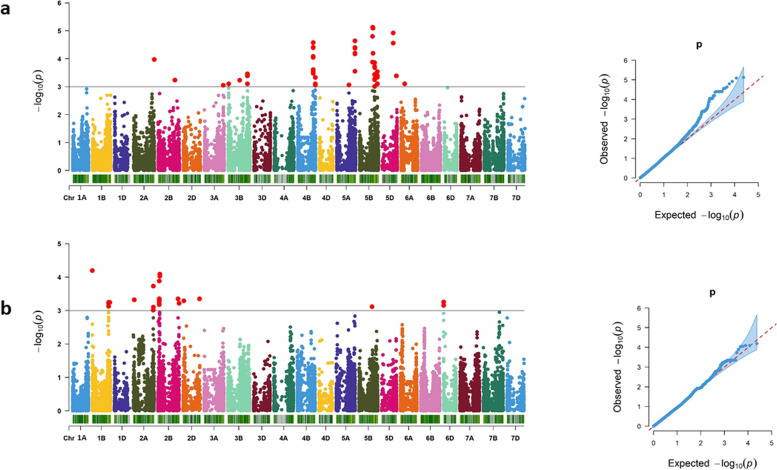
Manhattan and Q-Q plots of the GWAS on the STI of Pro and H_2_O_2_. Manhattan plot (left) and Q-Q plot (right) of Pro STI **a**; Manhattan plot (left) and Q-Q plot (right) of H_2_O_2_ STI **b**. − log_10_ (*P*) =3.0 is the significant threshold level for MTA and marked by a horizontal grey line in the Manhattan plots. Red dots designate significant markers

Marker-trait association (MTA) analysis revealed a combined total of 125 markers that passed the significance threshhold [*P* = 0.001 or –log_10_ (*p*) = 3.0] for Pro content under drought stress, the STI, and Pro accumulation under control conditions. The significant MTAs were observed across different chromosomes (Figs. 2a, 3a; Supplementary Fig. S1a). Under drought conditions, 36 significant markers spread over chromosomes 1A, 3B, 4A, 5A, 6D, 6B and 7B, were identified for Pro content (Fig. 1a). These markers explained 3.41 to 5.51% of phenotypic variation (Tables 2, 3; Supplementary Table S5, S6). The top most significant marker, wsnp_Ex_rep_c106111_90308719 (*P* = 0.000003) located on chromosome 1A (8.23 Mbp), established a haplotype block (Pro_1A_Hap1). This chromosome harbored 9 significant markers. The hotspot region of significant markers for Pro content under drought stress was located on chromosome 4A and comprised of 12 markers across 41.95 to 46.12 Mbp. We observed that 19 SNPs out of 36 overlapped with candidate genes that possessed 52% of significant SNPs.

**Table 2:** Marker, chromosome (Chr), position, P-value, phenotypic variation (PV), allele, favorable (Fav.) allele, T-test value regarding Pro and H_2_O_2_ accumulation under drought stress and for the STI of Pro and H_2_O_2_

Trait	Marker	Chr	Position	P-value	PV (%)	Allele (Major: Minor)	Fav. allele	T-test value
Pro_dro	RFL_Contig1027_442	1A	8,244,106	3.48E-05	5.51	A:G	G	0.02
	AX-158569423	1A	8,248,738	8.81E-05	5.0	G:A	A	0.03
	BS00084022_51	1A	31,781,700	9.57E-04	3.42	T:C	T	0.02
	CAP12_rep_c3868_270	3B	986,619	9.42E-04	3.41	C:A	A	0.03
	AX-158541844	3B	20,538,743	6.95E-05	5.11	G:T	T	< 0.01
	AX-158538340	3B	20,717,449	8.92E-04	3.52	A:G	G	< 0.01
	AX-158541844	3B	20,538,743	6.95E-05	5.12	G:T	T	< 0.01
	AX-158538340	3B	20,717,449	8.92E-04	3.51	A:G	G	< 0.01
	AX-158541845	3B	20,718,475	1.60E-04	4.72	G:A	A	0.01
	AX-111526074	3B	20,719,624	1.60E-04	4.71	G:A	A	< 0.01
	BS00009970_51	4A	45,338,226	3.46E-04	4.13	T:C	T	< 0.01
	AX-111497637	4A	46,292,909	5.82E-05	5.21	C: T	C	< 0.01
	wsnp_Ex_c57094_58953404	5A	9,657,523	3.30E-04	4.11	A:G	G	< 0.01
	Excalibur_c2991_320	6D	469,919,670	1.25E-04	4.62	C:T	C	< 0.01
Pro_STI	AX-110412102	2A	775,936,337	1.07E-04	9.42	A:G	G	< 0.01
	AX-158540981	2B	632,394,417	5.82E-04	6.73	A:G	G	< 0.01
	AX-158523479	3A	712,134,412	8.94E-04	6.33	C:T	T	< 0.01
	BS00003522_51	3B	432,844,940	5.91E-04	6.72	A:G	G	< 0.01
	Ku_c1575_338	3B	726,481,592	7.93E-04	7.41	G:A	G	0.02
	AX-158550762	5A	451,456,903	8.66E-04	6.32	T:C	T	0.03
	Jagger_c3991_101	5B	488,820,722	1.33E-04	8.33	T:C	C	< 0.01
	GENE_3437_148	5B	489,280,672	1.59E-05	10.81	T:C	C	< 0.01
	AX-158525047	5B	490,619,499	7.52E-06	12.12	C:T	T	< 0.01
H_2_O_2__dro	AX-158557366	2A	749,105,336	3.95E-05	10.17	A:G	G	< 0.01
	wsnp_JD_c9360_10216330	3B	610,751,737	6.08E-04	6.82	C:T	T	< 0.01
	AX-158557258	2A	751,013,994	8.12E-04	7.33	G:T	T	< 0.01
H_2_O_2__STI	AX-111649657	1B	614,277,801	8.74E-04	6.31	A:G	G	< 0.01
	AX-89670926	1B	614,293,304	6.68E-04	6.62	G:A	G	< 0.01
	AX-86183817	1B	614,452,475	6.50E-04	6.64	G:A	G	< 0.01
	AX-110434034	1B	614,612,411	5.83E-04	6.75	C:T	T	< 0.01
	AX-158602322	2A	34,152,277	5.20E-04	6.88	A:G	G	< 0.01
	AX-158596005	2A	751,004,758	8.52E-04	6.85	C:T	T	< 0.01
	Kukri_c30020_302	2A	751,094,015	2.10E-04	8.33	C:T	T	< 0.01
	Excalibur_c9752_289	2B	760,950,856	5.06E-04	6.91	C:T	T	< 0.01
	AX-158597023	2B	761,322,528	5.06E-04	6.91	C:T	T	< 0.01
	AX-158596842	2B	800,060,795	4.34E-04	7.23	G:A	A	< 0.01
	BobWhite_c11059_169	2D	32,053,537	5.65E-04	6.82	A:C	C	< 0.01
	Tdurum_contig42636_995	5B	491,957,849	9.70E-04	6.22	C:T	T	< 0.01

Abbreviations: *Pro_dro,* Proline content under drought conditions; *H*_*2*_*O*_*2_*_*dro,* H_2_O_2_ content under drought conditions

**Table 3:** Haplotype blocks, number of markers in haplotype block (NMHB), chromosome (Chr), haplotype block (HB) size, haplotype allele and favorable allele regarding Pro and H_2_O_2_ accumulation under drought stress and for the STI

Trait	Haplotype block	NMHB	Chr	HB size (bp)	Haplotype alleles (Ma: Mi)	Favorable allele
Pro_dro	Pro_1A_Hap1	3	1A	404,126	TGT: CTC	CTC
	Pro_1A_Hap2	6	1A	10,436	ACCTGG: GTTGGT	GTTGGT
	Pro_1A_Hap3	6	1A	197,978	CCAAAT: CCGGCT	CCAAAT
	Pro_3B_Hap1	4	3B	1281	GGCG: AGCG	AGCG
	Pro_3B_Hap2	6	3B	197,149	CGCCCG: CGTCCG	CGTCCG
	Pro_4A_Hap1	13	4A	2,587,578	GCCTAATCCTGTC: GCCTAATCTTGTC	GCCTAATCCTGTC
	Pro_4A_Hap2	4	4A	75,535	CCTA: TTCG	TTCG
	Pro_4A_Hap3	5	4A	1,780,008	ACTTG: GACCA	ACTTG
	Pro_6D_Hap1	3	6D	292,341	CGT: TAC	TAC
	Pro_7B_Hap1	4	7B	631,054	CTAG: CTGA	CTGA
Pro_STI	Pro_3B_Hap3	3	3B	2175	AGG: GGA	GGA
	Pro_3B_Hap4	7	3B	1,050,651	AGCATGC: CAAGCAC	AGCATGC
	Pro_4B_Hap1	20	4B	2,493,409	CACCCTACTCTGCGTATGTG: TGTTTCGTCTCTTACGCTCA	CACCCTACTCTGCGTATGTG
	Pro_4B_Hap2	8	4B	332,843	AAATTATG: CGGCCGCA	AAATTATG
	Pro_5A_Hap1	11	5A	408,220	CGCCATTAACG: TATAGCCGGTA	CGCCATTAACG
	Pro_5B_Hap1	6	5B	787,764	GCCCAC: ATATGC	ATATGC
	Pro_5B_Hap2	4	5B	284,390	GTGG: TGAA	TGAA
	Pro_5B_Hap3	8	5B	2,005,360	AGCGTAAT: CATAGCGG	CATAGCGG
	Pro_5B_Hap4	8	5B	2,619,512	CGACGACC: TAGTGGTT	TAGTGGTT
	Pro_5B_Hap5	4	5B	7048	GGAT: AGGC	AGGC
	Pro_5B_Hap6	18	5B	3,739,633	CAATCATATATCTCCAAG: TGGCCATCCGCTCATCGG	TGGCCATCCGCTCATCGG
	Pro_5B_Hap7	18	5B	933,082	CTCCCCCGCTGTAACACA: CTCCCCCGCTGTAACATA	CTCCCCCGCTGTAACACA
H_2_O_2__ STI	HP___1B_Hap1	3	1B	24,816	GTA: ACG	ACG
	HP___1B_Hap2	3	1B	9056	CGA: AAG	AAG
	HP ___2B_Hap1	11	2B	2,193,600	GCGCTGTTTGT: ATATCACCCAT	ATATCACCCAT
	HP ___2B_Hap2	6	2B	3,877,092	AATCCG: CATCCG	AATCCG
	HP ___2D_Hap1	3	2D	1,304,253	ACC: GTC	GTC
	HP ___6D_Hap1	4	6D	21,999	ACCC: GTTT	GTTT

Abbreviations: *Pro_dro,* Proline content under drought conditions

For the STI of Pro, 71 significant markers were identified, which explained 6.30 to 12.12% of phenotypic variation (Tables 2, 3; Supplementary Table 5, 6). The topmost significant marker AX-158525047 (*P* = 0.000008), located on chromosome 5B (490.61 Mbp), showed the highest phenotypic variation among all significant markers (12.12%) (Fig. 3a; Table 2). Other MTAs were identified on chromosomes 2A, 2B, 3A, 3B, 4B, 5A, 5B, 5D and 6A. The hotspot region for the STI of Pro was identified on chromosome 5B which contained 30 significant markers within the span of 490.61 Mbp to 565.76 Mbp. We observed that 28% of SNPs overlapped with putative candidate genes (Table 2).

Under control conditions, 18 significant markers were associated with Pro. MTAs were observed on chromosomes 1A, 2A, 3B, 4A, 4B, 5A, 5B, 5D, 6B, 7B. These markers accounted for 3.33 to 4.26% of phenotypic variation (Tables 2, 3; Supplementary Table S5, S6). AX-158524974 located at 5B was the most significant marker (*P* = 0.0002) and accounted for 4.26% of phenotypic variation (Supplementary Table S5). A hotspot region with 4 significant markers was located on chromosome 5B (588.51 to 712.60 Mbp). For H_2_O_2,_ a total of 53 significant markers, including 16 markers for control, 2 for drought and 34 for the STI, were identified by MTA analysis (Tables 2, 3; Supplementary Table S5, S6). Under drought conditions, H_2_O_2_ accumulation is associated with 2 significant markers. The most significant marker was AX-158557366 (*P* = 0.00004) located on chromosome 2A (749.10 Mbp). It exhibited 10.17% phenotypic variation (Fig. 2b; Table 2). Another significant marker, wsnp_JD_c9360_10216330 (*P* = 0.0007) was located on chromosome 3B (610.75 Mbp).

For the STI of H_2_O_2,_ the association study identified MTAs across chromosomes 1B, 2A, 2B, 2D, 5B and 6D (Fig. 3b). Those markers were responsible for 6.22 to 8.33% of phenotypic variation (Tables 2, 3; Supplementary Table S5, S6). The highest signal was observed on chromosome 1B at 43.20 Mbp. The marker Kukri_c79308_278 (*P* = 0.00008) was the most significant marker and formed a haplotype block (HP_1B_Hap1) (Table 3). A hotspot region for significant markers was located on chromosome 2B and consists of 19 markers covering the region between 51.92 Mbp and 800.06 Mbp. We observed that 32 out of 57 markers overlapped with putative candidate genes (either introns or exons), which comprised 56% of significant SNPs (Supplementary Table S8b).

For H_2_O_2_ content under control conditions_,_ significant markers were identified on chromosomes 1B, 2A, 2B, 2D, 6B and 6D. Those markers caused 7.0 to 8.22% of phenotypic variation (Tables 2, 3; Supplementary Table S5, S6). The most significant SNP, Kukri_c79308_278 (*P* = 0.00006), was located on chromosome 1B (4.32 Mbp) and formed a haplotype block (HP___1B_Hap1) (Table 3). In total, 12 markers were localized on chromosome 2B; therefore, it was recognized as the hotspot region for markers associated with H_2_O_2_ accumulation under control conditions. Overall, our observation revealed that significant MTAs were most prevalent in the A genome followed by the B and D genomes, respectively.

### Major and minor alleles of significant markers showed variable associations with pro and H_2_O_2_ content

Contrasting alleles of a trait are important sources for plant breeding programs. Initially, we observed significant markers either in established linkage disequilibrium (LD) blocks with nearby markers or on their own, then we estimated their allelic effect (Tables 2, 3; Supplementary Table S5, S6). LD analysis revealed that out of 125 SNPs 89 formed 25 haplotype blocks, while 36 markers remained individual. Under drought conditions, significant markers for Pro accumulation on chromosomes 1A, 3B, 4A, 6D and 7B formed 3, 2, 3, 1 and 1 haplotype blocks, respectively (Table 3). Among them, the biggest haplotype block, Pro_4A_Hap1, was found on chromosome 4A and comprised of 13 markers. Under control conditions, significant markers associated with Pro content formed one haplotype block in each of chromosomes 1A, 5A and 7B (Supplementary Table S6). For the STI of Pro, haplotype blocks were formed on chromosomes 3B, 4B, 5A and 5B. Pro_4B_Hap1, a haplotype block on 4B chromosome, was revealed as the largest block (2.49 Mbp), which contained 20 markers (Table 3).

Variation of H_2_O_2_ accumulation under drought conditions was only associated with the top most significant SNP, AX-158557366 located on chromosome 2A. For the STI of H_2_O_2_, 6 haplotype blocks were detected on chromosomes 1B, 2B, 2D and 6D (Table 3). H_2_O_2_ content under control conditions was linked with 3 haplotype blocks across chromosomes 1B, 2B and 6B. Among them, HP_2B_Hap1 was the largest haplotype block (2.19 Mbp). LD analysis showed that 70, 77 and 100% of minor alleles of single-markers and 77, 58 and 61.63% of haplotypes were associated with high Pro content under drought stress, the STI of Pro and Pro content under control conditions, respectively. Under drought conditions, H_2_O_2_ content was associated with the topmost significant SNP AX-158557366 (Table 2). We observed more than 60% of minor alleles of single-markers and haplotypes were associated with a high STI and H_2_O_2_ content under control conditions. In general, our findings showed that the minor alleles of haplotypes and single-markers were linked with higher Pro and H_2_O_2_ content and with the high STI of Pro and H_2_O_2_.

### Markers pleiotropy identified common markers between traits

To identify pleiotropic markers, loci that were linked to several traits were considered. This way, we recognized 12 pleiotropic SNPs. The SNP at locus AX-111526074 on chromosome 3B was connected to the STI of Pro and Pro accumulation under drought conditions. Similarly, the markers Kukri_c79308_278 and AX-158602322 located on chromosomes 1B and 2A, respectively, and the markers AX-158575274, AX-158547448, AX-158597348, BS00009807_51, IAAV3165, Kukri_c37311_136, wsnp_Ex_c10596_17293192, and wsnp_Ex_c10596_17293363 all located on chromosome 2B were related to Pro content under control conditions and the STI of H_2_O_2._ Finally, the marker RFL_Contig1027_442, associated with Pro content under drought stress, was identified as pleiotropic. It was previously associated with yield by [27]. There was no marker that linked to both Pro and H_2_O_2_ accumulation under drought conditions.

### Candidate loci harbored putative candidate genes

Candidate gene analysis was performed to find genes potentially involved in Pro and H_2_O_2_ accumulation under drought stress and the STI of Pro and H_2_O_2_. To create a short list of putative candidate genes, all genes (promoter, exon or intron regions) that overlapped with significant SNPs or haplotype block regions were retrieved (Table 4). 1 Mb regions of upstream and downstream of significant SNP loci were scanned to find more candidate genes that are provided in Supplementary Table S8a and S8b. The two most significant single SNPs for Pro content under drought conditions, RFL_Contig1027_442 and AX-158569423, coincided with the gene *TraesCS1A01G015300* that encodes for a Ras-like protein (Table 4). AX-158569423 was located in the 4th intron of *TraesCS1A01G015300*. About 90% (*n* = 155) of the screened cultivars possessed ‘G’ alleles of *TraesCS1A01G015300*. This major allele showed significantly less Pro accumulation compared to the minor allele (Table 2 and Supplementary Fig. S2a). The third significant single SNP was linked with a CaM binding domain-containing protein. Three haplotype blocks on chromosome 1A were linked with Pro accumulation under drought stress; among them, Pro_1A_Hap1 was linked with four serine/threonine-protein kinase encoding genes(GO: 0004672) (Table 4). The major haplotype of this block ‘TGT’ was linked with lower Pro content. 72% of the examined cultivars (*n* = 116) possessed it, whereas the haplotype ‘CTC’ was identified as favorable but possessed by only 11% of the cultivars (*n* = 17) (Supplementary Fig. S2b). Pro_1A_Hap2 was associated with a gene coding for a transmembrane protein. The haplotype region of Pro_1A_Hap3 coincided with F-box family protein-coding genes and the haplotype Pro_3B_Hap1 was associated with a MYB transcription factor and a NBS-LRR disease resistance gene. Significant markers underlying the Pro_4A_Hap1 block were associated with genes encoding a protein kinase (*TraesCS4A01G049900*), a phosphatase 2C family protein (*TraesCS4A01G050000*) and tetratricopeptide repeat proteins (*TraesCS4A01G050100* and *TraesCS4A01G050400*). These genes are involved in protein binding (GO: 0005515), DNA binding (GO: 0003677) and adenosine diphosphate (ADP) binding (GO: 0043531) activities. The haplotype Pro_6D_Hap1 was associated with the *TraesCS6D01G401100* gene encoding a pentatricopeptide repeat-containing protein with protein binding (GO: 0005515) activity. The haplotype Pro_7B_Hap1 coincided with a zinc finger family protein (*TraesCS7B01G122900*) and a receptor-like protein kinase (*TraesCS7B01G122800*). Moreover, several F box family protein genes were observed in other significant SNPs for Pro content under drought conditions (Table 4 and Supplemental Table S8a).

**Table 4:** A short-list of putative candidate genes related to Pro and H_2_O_2_ accumulation under drought stress and theSTI. The genes overlap with the significant markers or haplotype block regions

Trait	Marker/Haplotype	Chr	Position (bp)	Candidate gene	Protein	Gene OntolGene Ontology
Pro_dro	AX-158569423	1A	8,248,738	*TraesCS1A01G015300*	Ras-like protein	GO:0005525 MF: GTP binding
	BS00009970_51	4A	45,338,226	*TraesCS4A01G053700*	CaM_binding domain-containing protein	
	Pro_1A_Hap1	1A	7,835,834 to 8,239,960	*TraesCS1A01G014400*	Serine/threonine-protein kinase	GO:0004672 MF: protein kinase activity
				*TraesCS1A01G014500*	Serine/threonine-protein kinase	GO:0004672 MF: protein kinase activity
				*TraesCS1A01G014900*	Serine/threonine-protein kinase	GO:0004672 MF: protein kinase activity
				*TraesCS1A01G015000*	Serine/threonine-protein kinase	GO:0004672 MF: protein kinase activity
	Pro_1A_Hap2	1A	33,365,205 to 33,375,641	*TraesCS1A01G051900*	transmembrane protein	NA
	Pro_1A_Hap3	1A	586,717,462 to 586,915,440	*TraesCS1A01G051000*	F-box protein	NA
				*TraesCS1A01G051100*	F-box/LRR-repeat protein	NA
				*TraesCS1A01G051600*	F-box family protein	GO:0005515 MF: protein binding
	Pro_3B_Hap1	3B	18,820,910 to18822191	*TraesCS3B01G039100*	MYB transcription factor	GO:0003677 MF: DNA binding
				*TraesCS3B01G039200*	NBS-LRR disease resistance protein	GO:0043531 MF: ADP binding
	Pro_7B_Hap1	7B	144,074,930 to 144,705,984	*TraesCS7B01G122600*	Aquaporin	GO:0005215 MF: transporter activity
				*TraesCS7B01G122800*	Receptor-like protein kinase	GO:0004672 MF: protein kinase activity
				*TraesCS7B01G122900*	Zinc finger family protein	GO:0003676 MF: nucleic acid binding
	Pro_6D_Hap1	6D	470,391,193 to 470,683,534	*TraesCS6D01G401100*	Pentatricopeptide repeat-containing protein	GO:0005515 MF: protein binding
	Pro_4A_Hap1	4A	40,442,320 to 43,031,174	*TraesCS4A01G049900*	Protein kinase	GO:0004672 MF: protein kinase activity
				*TraesCS4A01G050000*	Phosphatase 2C family protein	GO:0003824 MF: catalytic activity
				*TraesCS4A01G050100*	Tetratricopeptide repeat protein 1	GO:0005515 MF: protein binding
				*TraesCS4A01G050400*	Pentatricopeptide repeat-containing protein, putative	GO:0005515 MF: protein binding
				*TraesCS4A01G050500*	Metal transporter	GO:0005215 MF: transporter activity
Pro_STI	AX-110412102	2A	775,936,337	*TraesCS2A01G584400*	WAT1-related protein	GO:0016020 CC: membrane
	Ku_c1575_338	3B	726,481,592	*TraesCS3B01G475800*	Auxin response factor	GO:0003677 MF: DNA binding
	AX-158550762	5A	451,456,903	*TraesCS5A01G235300*	Hexosyltransferase	GO:0016758
	AX-158525047	5B	490,619,499	*TraesCS5B01G307200*	DNA polymerase delta subunit 4	GO:0006260 BP: DNA replication
	GENE_3437_148	5B	489,280,672	*TraesCS5B01G305100*	PF02181: Formin Homology 2 Domain	G
	Jagger_c3991_101	5B	488,820,722	*TraesCS5B01G304800*	cytochrome P450, family 702, subfamily A, polypeptide 6	NA
	Excalibur_c9846_458	5B	505,482,207 to 506,269,971	*TraesCS5B01G320600*	Pentatricopeptide repeat-containing protein	GO:0005515 MF: protein binding
	Pro_3B_Hap4	3B		*TraesCS3B01G475800*	Auxin response factor	GO:0003677 MF: DNA binding
				*TraesCS3B01G476500*	F-box protein	NA
				*TraesCS3B01G476700*	Auxin response factor	NA
				*TraesCS3B01G476800*	Prefoldin subunit 5	NA
				*TraesCS3B01G476900*	F-box protein family	GO:0005515 MF: protein binding
				*TraesCS3B01G477000*	E3 ubiquitin protein ligase drip2	NA
	Pro_4B_Hap1	4B		*TraesCS4B01G292700*	Transmembrane protein, putative	NA
				*TraesCS4B01G292900*	AP2-like ethylene-responsive transcription factor	GO:0003677 MF: DNA binding
	Pro_5A_Hap1	5A		*TraesCS5A01G516800*	Basic-leucine zipper (bZIP) transcription factor family protein	NA
	Pro_5B_Hap1	5B		*TraesCS5B01G320600*	Pentatricopeptide repeat-containing protein	GO:0005515 MF: protein binding
	Pro_5B_Hap2	5B		*TraesCS5B01G321800*	Uncharacterized protein	GO:0005524 MF: ATP binding
	Pro_5B_Hap3	5B		*TraesCS5B01G387400*	F-box family protein	GO:0005515 MF: protein binding
H_2_O_2__STI	HP___1B_Hap1	1B		*TraesCS1B01G007800*	TAF domain-containing protein	GO:0046982 MF: protein heterodimerization
	AX-86183817	1B	614,452,475	*TraesCS1B01G382000*	Lys-63-specific deubiquitinase BRCC36	GO:0005515 MF: protein binding
	AX-158596005	2A	751,004,758	*TraesCS2A01G537100*	Superoxide dismutase	GO:0004784 MF: superoxide dismutase activity
	Excalibur_c9752_289	2B	760,950,856	*TraesCS2B01G570500*	phosphatidylinositol 4-kinase gamma-like protein	GO:0016301 MF: kinase activity
	HP _2B_Hap1	2B	51,928,949 to 73,198,699	*TraesCS2B01G090200*	F-box family protein	GO:0005515 MF: protein binding
				*TraesCS2B01G091500*	Cytochrome P450	GO:0005506 MF: iron ion binding
				*TraesCS2B01G566900*	Serine/threonine-protein kinase	GO:0004672 MF: protein kinase activity
	HP ___2B_Hap2	2B	758,593,004 to 760,933,257	*TraesCS2B01G567000*	Serine/threonine-protein kinase	GO:0004672 MF: protein kinase activity
				*TraesCS2B01G567100*	Serine/threonine-protein kinase	GO:0004674 MF: protein serine/threonine kinase activity
				*TraesCS2B01G567500*	Serine/threonine-protein kinase	GO:0004672 MF: protein kinase activity
				*TraesCS2B01G567600*	Superoxide dismutase	GO:0004784 MF: superoxide dismutase activity
				*TraesCS2B01G567700*	Serine/threonine-protein kinase	GO:0004674 MF: protein serine/threonine kinase activity
				*TraesCS2B01G567800*	Serine/threonine-protein kinase	GO:0004672 MF: protein kinase activity
				*TraesCS2B01G568000*	Serine/threonine-protein kinase	GO:0004672 MF: protein kinase activity
				*TraesCS2B01G568500*	Serine/threonine-protein kinase	GO:0004672 MF: protein kinase activity
				*TraesCS2B01G568600*	Serine/threonine-protein kinase	GO:0004672 MF: protein kinase activity
	HP ___6D_Hap1	6D	3,119,406 to 3,141,405	*TraesCS6D01G007800*	receptor kinase 1	GO:0004672 MF: protein kinase activity
				*TraesCS6B01G138800*	F-box plant-like protein, putative	NA
				*TraesCS6B01G138900*	F-box plant-like protein, putative	NA
H_2_O_2__dro	AX-158557366	2A	749,105,336	*TraesCS2A01G533200*	Flavin-containing monooxygenase	GO:0016491 MF: oxidoreductase activity

Abbreviations: MF, molecular function; *Pro_dro,* Proline content under drought conditions; *H*_*2*_*O*_*2_*_*dro,* H_2_O_2_ content under drought conditions

In the case of the STI of Pro, a total of 56 protein-coding genes overlapped with significant SNPs and haplotype blocks (Supplementary Table S8a). The most significant marker, AX-158525047, coincided (53 base pair upstream of the ATG site) with the DNA polymerase delta subunit 4 (*TraesCS5B01G307200*) located on chromosome 5B. The second most significant marker was located within a haplotype block(Pro_5B_Hap2) on the same chromosome. This block was nested in the promoter region of *TraesCS5B01G321800* (183 base pairs upstream) encoding a protein with ATPase function (GO: 0005524). Haplotype analyses identified minor alleles of both AX-158525047 and Pro_5B_Hap2 represented by about 8% (*n* = 14) of the cultivars and linked with high STI values compared to the other alleles (Table 2 and Supplementary Fig. S3a-b). Other significant SNPs on chromosome 5B were linked with formin-like (*TraesCS5B01G305100*) and cytochrome P450 (*TraesCS5B01G304800*) proteins. The significant marker locus AX-110412102 coincided with the WAT1-related protein coding gene *TraesCS2A01G584400*. Another significant SNP, AX-110412102, overlapped with an auxin response factor (*TraesCS3B01G475800*). Putative candidate genes linked with other haplotypes encode an F-box protein, (*TraesCS4B01G292900*), a basic-leucine zipper (bZIP) transcription factor family protein (*TraesCS5A01G516800*) and a pentatricopeptide repeat-containing protein (*TraesCS5B01G320600*) (Table 4). Several candidate genes were also identified in the 1 Mb up- and downstream regions of significant SNPs. They encode, for example, Lys-63-specific deubiquitinase, superoxide dismutase, sulfotransferase proteins and ethylene-responsive transcription factors. Additionally, the homologs and orthologs analysis of these genes in *Arabidopsis*. identified no genes as homologs in Arabidopsis (Supplementary Table S9).

H_2_O_2_ content under drought stress was associated with top most significant SNP AX-158557366 which coincided with a gene that encodes a flavin-containing monooxygenase (*TraesCS2A01G533200)* with oxidoreductase activity (Table 4). The gene *TraesCS2A01G533200* harbored the SNP AX-158557366 in the promoter region (697 bp downstream). The minor allele (*n* = 19) of this gene was linked with high H_2_O_2_ content (Supplementary Fig. S4a). In the case of the STI of H_2_O_2_, the significant marker AX-86183817 encompassed a Lys-63-specific deubiquitinase protein-coding gene. The significant marker AX-158596005 located on chromosome 2A was linked with the gene *TraesCS2A01G537100* that encodes superoxide dismutase (Table 4). This SNP was located within the first exon of *TraesCS2A01G537100*. The allelic variation among the population revealed ‘T’ to be the minor allele (*n* = 15) associated with high STI (Table 2 and Supplementary Fig. S4b). Among the haplotype block regions, HP_2B_Hap2 covered nine serine/threonine-protein kinase genes and one superoxide dismutase protein-coding. Several putative candidate genes coding for disease resistance proteins and zinc finger, pentatricopeptide repeat-containing proteins were identified within 1 Mb up- and downstream of significant SNPs (Supplementary Table S8b). Overall, the candidate gene analysis found several genes linked with Pro and H_2_O_2_ accumulation under drought conditions and the STI of Pro and H_2_O_2_.

## Discussion

### Diversity panel exhibits augmented phenotypic variation for pro and H_2_O_2_ in response to drought stress

Genetic diversity among the cultivars of a species is an important criterion asset for plant breeding research, especially to develop drought-tolerant wheat varieties [28]. The present study on wheat showed phenotypic variability of drought-induced Pro and H_2_O_2_ accumulation in a field environment which was not available before. We observed clear plant-to-plant Pro and H_2_O_2_ differences under both control and drought conditions, although the variation was wider in response to stress. The effect of genotype and environment interaction was significant, which indicates that the drought treatment enhanced Pro accumulation. A continuous phenotypic variation was also observed for both Pro and H_2_O_2_ content indicating polygenic inheritance. Pro accumulation was significantly different between modern and traditional sub-groups. The modern cultivars accumulated noticeably lower Pro amounts than traditional ones. Moreover, the variation of Pro accumulation under drought stress was higher in the traditional sub-group. In case of the STI of H_2_O_2_, the European group exhibited significantly higher values than the non-European sub-group. Our findings are in agreement with a previous report by [29], which identified a distinct LD decay among sub-populations of barley. In our case a distinct pattern of LD decay across the three genomes exist between the European and non-European as well as the traditional and modern sub-groups. Our results also indicate that the European sub-group is more responsive to drought stress than the non-European group, although an adaptive role needs to be illustrated further.

### Pro and H_2_O_2_ accumulation under drought stress might be a physiological marker for screening the cultivars and genetic improvement

Pro accumulation is known to correlate with drought stress [30]. Several studies regarding genetic [31], transcriptomic and proteomic analyses [32, 33] highlighted the importance of Pro in stress tolerance. Housekeeping amounts of Pro have been associated with signaling pathways of plant developmental and maturation processes that lead to enhanced vegetative growth and grain yield [34, 35]. H_2_O_2_ is regarded as a regulator of different stress response mechanisms [36]. Many signal transduction pathways are also triggered by H_2_O_2_ into plant cells under drought conditions. The correlation of Pro or H_2_O_2_ and yield attributes was not strong in our study. Therefore, our results suggest that Pro and H_2_O_2_ accumulation in leaves under drought stress might not be a direct determinant of yield attributes. It might rather have an adaptive function as a general indicator of drought. Compared to the control treatment, we observed an 11-fold increase of Pro content under drought conditions. This finding is similar to a previous study by [8], who reported Pro accumulation to be 100 times higher under drought condition as opposed to control conditions. Studies on barley [37] and wheat [38] reported that epidermis and vascular bundles had preferentially higher Pro content under stress conditions. These evidences support our results and suggest that Pro and H_2_O_2_ can be reliable markers for the assessment of wheat cultivars under drought stress.

Moreover, we identified contrasting alleles for the STI of H_2_O_2_ and Pro, which represents relative performance of each cultivar in response to drought. We found that minor alleles of significant markers are linked significantly with higher Pro and H_2_O_2_ content under drought stress compared to the major alleles (Tables 2, 3). For Pro, the ‘C’ allele of the most significant marker contributes to a lower STI, whereas cultivars with the ‘T’ allele showed higher STIs (Supplementary Table S3). The cultivar ‘Akteur’ displayed the lowest STI of Pro and ‘Centurk’ the highest. Similarly, the most significant marker for the STI of H_2_O_2_ is a haplotype of which the ‘GTA’ allele is linked with a low and the ‘ACG’ with a high STI. The cultivar ‘Urban’ had the lowest STI of H_2_O_2_, while‘Mironovs’ possessed highest. These results indicate that drought induces Pro and H_2_O_2_ accumulation. Collectively, our findings reveal that Pro and H_2_O_2_ accumulation under drought stress might be a physiological marker in plants and the contrasting alleles could be utilized for marker-assisted breeding programs.

### GWAS identifies candidate loci and genes

Dissecting the genetic regulators underlying drought-induced Pro and H_2_O_2_ variations is one of the prime targets for further functional studies. We revealed a large genetic diversity within the population which is important for GWAS and further genetic studies. According to a previous study [39], the natural variation among drought-related traits helps to identify the best resources for genetic studies. In this study, a GWAS was employed to identify candidate loci associated with Pro and H_2_O_2_ variation in response to drought. The identified loci were associated to several candidate genes possibly involved in Pro- and H_2_O_2_-mediated stress responses. The two most significant markers linked to Pro content under drought conditions coincided with a gene that encodes a Ras-like GTP-binding protein. Ras proteins play a pivotal role in signal transduction. The ortholog in *Arabidopsis* has been identified as a drought-responsive gene and its over expression was associated with drought tolerance [40]. Transmembrane protein (TP) coding genes have been identified in a few haplotype block regions. Reportedly, TPs are involved in Pro transport in different species [41, 42]. The gene of a protein with a Calmodulin (CaM) binding domain was found to be linked with significant SNPs. CaM is a ubiquitous calcium-binding protein that can regulate diverse cellular functions by modulating the activity of various enzymes and proteins. This gene has been recorded to improve stress tolerance by facilitating Pro accumulation [43]. All the evidence suggests that the candidate genes encodingRas proteins, TPs and CaM binding domain proteins contribute to the variation of Pro accumulation under drought stress.

A few haplotypes associated with the STI of Pro are linked to genes of F-box proteins. In a previous report, the overexpression of an F-box protein gene in tobacco improved stress tolerance [44]. F-Box genes have also been associated with Pro content in wheat [45]. Therefore, it is reasonable to assume that the candidate F-box genes in this study might be involved in the Pro metabolism. More haplotypes were associated with the gene *TraesCS4B01G292900* (AP2-like ethylene-responsive transcription factor). AP2/ERF is one of the ideal candidates for crop improvement since its overexpression in plants was shown to enhance tolerance to drought, salt and freezing [46, 47]. A recent GWAS identified that ethylene-responsive transcription factors are involved in Pro metabolism in Eucalyptus [48]. Both the STI of Pro and H_2_O_2_ were related to Cytochrome P450 proteins. These are known to produce H_2_O_2_ and play an important role in stress tolerance [49]. Several candidate genes we identified encode for zinc finger proteins (ZFPs). ZFPs belong to a large protein family involved in Pro biosynthesis, stress responses and ROS scavenging mechanisms [50, 51]. Finally, several genes in our candidate loci regions encode for pentatricopeptide repeat and serine-threonine kinase proteins. These proteins have been reported as positive regulators of plant responses to abiotic stress and promote drought tolerance by increasing Pro accumulation [41, 52]. In a previous GWAS, for example, pentatricopeptide repeat and serine-threonine kinase protein-coding genes were linked with Pro metabolism in *Arabidopsis* [25]. Therefore, it stands to reason that pentatricopeptide repeat and serine-threonine kinase protein-coding genes might be involved in the Pro metabolism of wheat, too.

Top most significant SNP for H_2_O_2_ content under drought conditions was linked with the flavin-containing monooxygenase. Recently, flavin-containing monooxygenase has been identfied as a source of hydrogen peroxide [53]. The significant locus AX-158596005 was associated with the STI of H_2_O_2_ and encompasses a gene that encodes for superoxide dismutase (SOD). SOD is a ubiquitous antioxidant enzyme that converts the superoxide radical to H_2_O_2_. This protein has been shown to play a role in  drought tolerance [54]. Some significant SNPs for the STI of H_2_O_2_ are associated with disease resistance proteins. Disease resistance proteins are part of an adaptive machinery in plants that is involved in stress responses. In a previous study, N1P1 was identified as a disease resistance protein involved in H_2_O_2_ signaling [23]. The second most significant locus on the 1B chromosome for the STI of H_2_O_2_ is linked with the gene of the E3 ubiquitin-protein ligase ORTHRUS 2. This gene is involved in protein modification via the protein ubiquitination pathway [55]. revealed that H_2_O_2_ causes protein modifications, thereby, changing protein function. All this evidence support our findings and suggests that the identified candidate genes for H_2_O_2_ are the potential candidates that regulate H_2_O_2_ in response to the drought.

## Conclusions

Pro and H_2_O_2_ accumulation during stress conditions plays a multi-dimensional role for plant adaptation, therefore, studying the underlying genetic components is an important area of research. This study identified large genetic variation of drought-induced Pro and H_2_O_2_ accumulation. The traditional sub-group accumulated more Pro than cultivars the of modern sub-group under drought conditions. The European sub-group exhibited significantly higher STIs of H_2_O_2_ than the non-European. Our GWAS identified significant MTAs on different chromosomes for Pro and H_2_O_2_ under drought stress conditions and the STIs of Pro and H_2_O_2_. Minor alleles of single-markers and haplotypes are linked with higher Pro and H_2_O_2_ content under drought stress. Identified loci are reported for the first time in wheat under drought conditions. These loci and contrasting alleles are valuable resources for further functional studies and can be incorporated in marker-assisted breeding for cultivars with improved drought stress tolerance.

## Methods

### Plant material and experimental set up

The study was conducted with a global collection of 184 winter wheat cultivars. 60% of the cultivars originated from Germany while the remaining 40% came from the United States of America (USA), the United Kingdom, Mexico, France, Denmark, Serbia, Chile, Australia and Ukraine (Supplementary Table S1). The seeds were obtained from the Plant Breeding Division of the University of Bonn, Germany. Briefs of the cultivars are available in previous publication [27, 40]. The experiment was performed during the summer season 2019/2020 at campus Klein-Altendorf (50.4°N; 6.99°E; 160 m above sea level), the experimental station of the University of Bonn, Germany. The experimental set up followed a split-plot design where the treatments, control and drought were in main plots. Within the main treatments the cultivars were further sub-divided into two blocks following randomized complete block design (RCBD). About 25 seeds were sown into single rows in a randomized way. The management and intercultural practices were followed according to a previous report [56]. Two sets of cultivars were prepared. One set was grown under the open field condition which is designated as “control treatment”, and another set was grown under a rain-out shelter, designated as “drought treatment”. Until the treatments began, both sets were watered and managed the same. The rain-out shelter had overhead sprinklers programmed to deliver ~ 5.00 mm water per day until the plants reached the heading stage (BBCH 51). Then, irrigation was stopped to initiate drought. After nine days, dehydration symptoms started to appear among the cultivars under drought stress, while the leaves of cultivars under control treatment remained normal. Finally, the penultimate leaves from three individuals of each cultivar from each block were polled together. Thus, two replications were made from two block which included a total of 6 individuals from each cultivar. The samples were wrapped in aluminum foil, flash frozen in liquid nitrogen and further stored at -80^০^C. The average moisture content of all experimental plots was determined with an EM50 Data Logger (ICT International) at a depth of 0–30 cm and presented in Supplementary Table S4.

### Pro and H_2_O_2_ determination

Pro was estimated according to [57] with minor modifications. In brief, ninhydrin reagent (2.5 g ninhydrin in 60 ml glacial acetic acid and 40 ml 6 M phosphoric acid) and 3% of sulphosalicylic acid were prepared freshly. Samples were crushed in liquid nitrogen and 90–100 mg of chilled powder was taken into a 2.0 ml microcentrifuge tube. Then, 1.5 ml of 3% sulphosalicylic acid were added, mixed and centrifuged at 12,000 g for 5 minutes. 200 μl of the supernatant were mixed with 200 μl acetic acid and 200 μl ninhydrin reagent. Next, the mixture was incubated at 95 °C for 60 minutes. After incubation, the reaction was immediately put on ice for 5 minutes to stop it. Then, 600 μl of pure toluene were mixed in and left at room temperature for 30 minutes. The absorbance of the chromatophore was recorded at a wavelength of 520 nm with 10 reads per well through a 96 well plate using a microplate reader (TECAN Infinite 200 Pro, TECAN Group Limited, Switzerland). The Pro content of the samples was determined based on a standard curve and expressed as μg/g fresh weight of the plant.

H_2_O_2_ was determined according to a previously described method [58] with some modifications. Leaf tissue was frozen in liquid nitrogen and ground into a fine powder, and 90–100 mg were transferred to a 2.0 ml microcentrifuge tube and homogenized in 500 μl of 0.1% trichloroacetic acid (TCA) before centrifugation at 12,000 g for 10 minutes. Then, 200 μl of the supernatant were mixed with 200 μl of 10 mM potassium phosphate buffer and 400 μl of 1 M potassium iodide in a new 2.0 ml microcentrifuge tube through vortexing. The sample absorbance was recorded at 390 nm using the same microplate reader as used for Pro. The H_2_O_2_ content of the samples was determined based on a standard curve and expressed as μg/g FW of the plant.

### Correlation analyses with pro, H_2_O_2_ and yield attributes

The average values of yield attributes such as grain yield (GY), plant dry biomass weight (PBW), shoot dry mass weight (SDW), spike number (SN), kernel number (KN) and thousand kernel weight (TKW) of the same association panel and research field were retrieved from a recent publication (Koua et al., [59], Supplemental Table 5), and average values of Pro and H_2_O_2_ were calculated in the present study. Then, analyses of the relationships between those yield attributes, proline and H_2_O_2_ content under drought stress and control conditions were performed. The pearson’s correlation coefficients (r) were calculated using R and a correlation table was made with the ‘corstars’ function of the ‘xtable’ package.

### Data analysis

Statistical analysis was performed wtith the statistical computing software R (version 3.5.1), especially the packages ‘nlme’ and ‘emmeans’, and Microsoft excel 2013. The maximum, minimum, mean, and coefficient of variation (CV %) were calculated for Pro and H_2_O_2_ content. To determine the treatment, genotype and their interaction on phenotypic traits, two-way ANOVA was applied using a mixed linear model (MLM) where genotype and replication as a random effect with the treatment was regarded as fixed effects [56]. The stress tolerance index (STI) was calculated using the following formula:

$$\textrm{STI}=\left(\textrm{Yp}\times \textrm{Ys}\right)/{\left(\textrm{Xp}\right)}^2;$$

where Ys = phenotypic value of a genotype under drought-stressed conditions; Yp = phenotypic value of a genotype under non-stressed conditions, and Xp = mean phenotypic value of genotypes under non-stressed conditions [60].

### Genome wide association studies

Pro and H_2_O_2_ content under control and drought conditions and the STI were used to perform GWAS. But to improve normativity of data, a square root transformation was applied to the H_2_O_2_ content before conducting the GWAS. A total of 24,216 SNP markers, covering 21 chromosomes of wheat, as described by previous publications [61, 62] were employed for the association study. To remove missing SNPs those have minor allele frequency (MAF) of < 5%, data imputation was performed in TASSEL 5.2 with LinkImpute (LD-kNNi) [63]. Association mapping was also performed in TASSEL 5.2 following a compressed MLM incorporating the population structures with five principal components together with a kinship matrix. The kinship matrix and principal components were used to avoid false positive associations and to correct the population structure [64]. After the Bonferroni correction, only two SNPs for Pro accumulation under drought condition would pass the stringent significance threshold that over-corrects the marker trait association, therefore, it was ignored [65]. In accordance with previous studies [66, 67], a *P*-value of 0.001 [−log_10_(*p*) = 3.0] was set as the significance threshold instead. SNPs that satisfied the threshold *P*-value were considered true positives and used for candidate gene search. The data was visualized as Manhattan plots using the R package ‘CMplot’.

### Linkage disequilibrium (LD) and haplotype analysis

Based on the significant markers identified in the GWAS, LD analysis was performed using Haploview 4.2 to define candidate loci/haplotype blocks [68]. A LD heat-map was generated based on confidence bounds of the *D′* values ranging between > 0.98 to 0.7 [69]. Generally, LD blocks harbor both significant SNPs and non-significant markers together. We considered the whole blocks for haplotype analysis. A student’s t-test was performed both for single significant markers and the haplotype alleles to compare statistical differences between alleles. The significant marker alleles that exhibited distinct STI of Pro and H_2_O_2_ are listed in Supplementary Table S3.

### Search for putative candidate genes

Genes (promoters, exons and introns) coinciding with the identified loci were selected as candidate genes. A comprehensive list is shown in Supplementary Table S8. The LD decay of this association panel was estimated at 19, 38, and 17.5 Mbp across the A, D, and B genome, respectively, in a recent study [70]. Based on this LD decay information and a previous study [26] with the same population panel, the significant SNPs do not belong to any LD block, 1.0 Mega base pair (Mbp) windows on both sides of them were asserted as regions for the putative candidate gene search. Gene annotations and gene ontologies (GO) were obtained from the International Wheat Genome Sequencing Consortium (IWGSC) of ‘Chinese Spring’ Ref Seq v1.0 in the Wheat URGI database (https://wheat-urgi.versailles.inra.fr) [71]. Candidate genes were further investigated using past literature to understand their possible functions. Analysis of orthologs between wheat and *Arabidopsis* was conducted with Triticeae-Gene Tribe through one to one selection [72] (http://wheat.cau.edu.cn/TGT/).

## Supplementary Information

**Additional file 1.**

**Additional file 2.**

**Additional file 3.**

## Data Availability

All data generated or analyzed in this study are available from the corresponding author on reasonable request.

## Abbreviations

**Pro**: Proline
**H**_**2**_**O**_**2**_: Hydrogen Peroxide
**P5C**: Pyrroline-5-Carboxylate
**ROS**: Reactive Oxygen Species
**MTA**: Marker-Trait Association
**GWAS**: Genome Wide Association Study
