# Supplementary material for: Pinpointing genomic loci for drought-induced proline and hydrogen peroxide accumulation in bread wheat under field conditions
Source: BMC Plant Biol. 2022 Dec 13;22:584. doi: 10.1186/s12870-022-03943-9 (PMC9746221; doi:10.1186/s12870-022-03943-9)
Supplement: Supplementary file 1 — Additional file 1. [file 12870_2022_3943_MOESM1_ESM.docx]

Supplementary Table S1 Brief description of the 184 wheat cultivars used in this study

| **SL No.** | **For Pro** | **For H_2_O_2_** | **Varieties** | **Year of registration** | **Origin** |
| --- | --- | --- | --- | --- | --- |
| 1 | TG001 | TG001 | Einstein | 2002 | UK |
| 2 | TG002 | TG002 | Oakley | 2006 | UK |
| 3 | TG003 | - | Jafet | 2008 | Germany |
| 4 | TG005 | TG005 | Rebell | 2013 | Germany |
| 5 | TG006 | - | Memory | 2013 | Germany |
| 6 | TG007 | - | Kurt | 2013 | Germany |
| 7 | TG008 | TG008 | Zappa | 2009 | Germany |
| 8 | TG009 | TG009 | Chevalie | 2006 | France |
| 9 | TG010 | TG010 | Gordian | 2013 | Germany |
| 10 | TG011 | TG011 | Mentor | 2012 | Germany |
| 11 | TG012 | TG012 | Meister | 2010 | Germany |
| 12 | TG013 | TG013 | KWS_Sant | 2010 | England |
| 13 | TG014 | TG014 | Brigand | 2002 | UK |
| 14 | TG015 | TG015 | Profilus | 2008 | Germany |
| 15 | TG016 | TG016 | Durin | 2006 | UK |
| 16 | TG018 | TG018 | Paroli | 2004 | Germany |
| 17 | TG020 | TG020 | Kronjuwe | 1980 | Czech Republic |
| 18 | TG021 | TG021 | Desamo | 2013 | Germany |
| 19 | TG022 | TG022 | Carenius | 2006 | Germany |
| 20 | TG023 | TG023 | Mulan | 2006 | Germany |
| 21 | TG025 | TG025 | Nelson | 2011 | Germany |
| 22 | TG027 | TG027 | Goetz | 1978 | France |
| 23 | TG028 | TG028 | Robigous | 2002 | England |
| 24 | TG029 | TG029 | Anapolis | 2013 | Germany |
| 25 | TG030 | TG030 | Solstice | 2001 | England |
| 26 | TG031 | TG031 | Biscay | 2000 | Germany |
| 27 | TG032 | TG032 | Capone | 2012 | Germany |
| 28 | TG033 | TG033 | Tabasco | 2008 | Germany |
| 29 | TG035 | TG035 | Cubus | 2002 | Germany |
| 30 | TG036 | - | Edward | 2013 | Germany |
| 31 | TG037 | TG037 | Famulus | 2010 | Germany |
| 32 | TG038 | TG038 | Dekan | 1999 | Germany |
| 33 | TG039 | - | SW_Toppe | 2002 | Germany |
| 34 | TG040 | TG040 | Matrix | 2010 | Germany |
| 35 | TG041 | TG041 | Jenga | 2007 | Germany |
| 36 | TG043 | - | TJB_990_15 | 2003 | UK |
| 37 | TG044 | TG044 | Forum | 2012 | Germany |
| 38 | TG045 | TG045 | Colonia | 2011 | Germany |
| 39 | TG046 | TG046 | Transit | 1994 | Germany |
| 40 | TG047 | TG047 | Potenzia | 2006 | Germany |
| 41 | TG048 | TG048 | Gaucho | 2005 | USA |
| 42 | TG049 | TG049 | Tarso | 1994 | Germany |
| 43 | TG050 | TG050 | Hermann | 2004 | Germany |
| 44 | TG051 | TG051 | Glaucus | 2011 | Germany |
| 45 | TG052 | TG052 | Tuareg | 2005 | Germany |
| 46 | TG053 | TG053 | Atomic | 2012 | Germany |
| 47 | TG054 | TG054 | Tobak | 2011 | Germany |
| 48 | TG055 | - | Pionier | 2013 | Germany |
| 49 | TG056 | TG056 | Manager | 2006 | Germany |
| 50 | TG058 | TG058 | Limes | 2003 | Germany |
| 51 | TG059 | TG059 | Ritmo | 1993 | Germany |
| 52 | TG060 | TG060 | Kalahari | 2010 | Germany |
| 53 | TG061 | - | Intro | 2011 | Germany |
| 54 | TG062 | TG062 | Oxal | 2010 | Germany |
| 55 | TG063 | - | Zobel | 2006 | Germany |
| 56 | TG065 | TG065 | Joker | 2012 | Germany |
| 57 | TG066 | - | Global | 2009 | Germany |
| 58 | TG067 | TG067 | Elixer | 2012 | Germany |
| 59 | TG068 | TG068 | Fedor | 2007 | Germany |
| 60 | TG069 | TG069 | Türkis | 2004 | Turkey |
| 61 | TG070 | TG070 | Skagen | 2006 | Germany |
| 62 | TG071 | TG071 | Greif | 1989 | Germany |
| 63 | TG072 | TG072 | Esket | 2007 | Germany |
| 64 | TG073 | TG073 | Primus | 2009 | Germany |
| 65 | TG074 | TG074 | Skalmeje | 2006 | Germany |
| 66 | TG076 | TG076 | Enorm | 2002 | Germany |
| 67 | TG078 | TG078 | Skater | 2000 | Germany |
| 68 | TG079 | TG079 | Brillant | 2005 | Germany |
| 69 | TG080 | - | Inspirat | 2007 | Germany |
| 70 | TG082 | TG082 | Ellvis | 2002 | Germany |
| 71 | TG084 | TG084 | Maris_Hu | 1975 | UK |
| 72 | TG085 | TG085 | SY_Ferry | 2012 | Germany |
| 73 | TG087 | TG087 | Sponsor | 1995 | France |
| 74 | TG088 | TG088 | Impressi | 2005 | Germany |
| 75 | TG089 | TG089 | Winnetou | 2002 | Germany |
| 76 | TG090 | TG090 | Toronto | 1990 | Germany |
| 77 | TG091 | TG091 | Torrild | 2005 | Germany |
| 78 | TG092 | TG092 | Contra | 1990 | Germany |
| 79 | TG093 | TG093 | Schamane | 2005 | Germany |
| 80 | TG094 | TG094 | Granada | 1980 | Germany |
| 81 | TG095 | TG095 | KWS_Coba | 2013 | Germany |
| 82 | TG096 | TG096 | Tommi | 2002 | Germany |
| 83 | TG097 | TG097 | Saturn | 1973 | Germany |
| 84 | TG098 | TG098 | Severin | 1980 | Belgium |
| 85 | TG099 | TG099 | JB_Asano | 2008 | Germany |
| 86 | TG100 | TG100 | Kerubino | 2004 | Czech Republic |
| 87 | TG101 | TG101 | Arktis | 2010 | Germany |
| 88 | TG102 | TG102 | Urban | 1980 | Germany |
| 89 | TG103 | TG103 | Orestis | 1988 | Germany |
| 90 | TG104 | TG104 | Flair | 1996 | Germany |
| 91 | TG105 | TG105 | Anthus | 2005 | Germany |
| 92 | TG106 | TG106 | Bombus | 2012 | Germany |
| 93 | TG107 | TG107 | Lucius | 2006 | Germany |
| 94 | TG108 | TG108 | Herzog | 1986 | Germany |
| 95 | TG109 | TG109 | Sorbas | 1985 | Germany |
| 96 | TG110 | TG110 | Tabor | 1979 | Germany |
| 97 | TG111 | - | Terrier | 2001 | Germany |
| 98 | TG112 | TG112 | Magister | 2005 | Germany |
| 99 | TG113 | TG113 | Altos | 2000 | Germany |
| 100 | TG114 | - | Progress | 2000 | France |
| 101 | TG116 | TG116 | Avenir | 2013 | Germany |
| 102 | TG117 | TG117 | Pantus | 1966 | Germany |
| 103 | TG118 | TG118 | Drifter | 1999 | Germany |
| 104 | TG120 | TG120 | Kranich | 2007 | Germany |
| 105 | TG121 | TG121 | Sperber | 1982 | Germany |
| 106 | TG123 | TG123 | Helios | 1980 | USA |
| 107 | TG124 | TG124 | Obelisk | 1987 | Netherlands |
| 108 | TG125 | TG125 | Magnus | 2000 | Germany |
| 109 | TG126 | - | Disponen | 1975 | Germany |
| 110 | TG127 | TG127 | Tambor | 1993 | Germany |
| 111 | TG128 | TG128 | Boxer | 2013 | Germany |
| 112 | TG129 | TG129 | Sokrates | 2001 | Germany |
| 113 | TG130 | TG130 | Carisupe | 1975 | Germany |
| 114 | TG131 | TG131 | Rektor | 1980 | Germany |
| 115 | TG132 | TG132 | Alves | 2010 | Germany |
| 116 | TG133 | TG133 | NaturaSt | 2002 | Germany |
| 117 | TG134 | TG134 | Alidos | 1987 | Germany |
| 118 | TG135 | TG135 | Monopol | 1975 | Germany |
| 119 | TG136 | TG136 | Akratos | 2004 | Germany |
| 120 | TG137 | TG137 | Knirps | 1985 | Germany |
| 121 | TG138 | TG138 | Bussard | 1990 | Germany |
| 122 | TG141 | TG141 | Tiger | 2001 | Germany |
| 123 | TG142 | TG142 | Ibis | 1991 | Chile |
| 124 | TG143 | TG143 | Batis | 1994 | Czech Republic |
| 125 | TG144 | TG144 | Topfit | 1972 | Germany |
| 126 | TG145 | TG145 | Akteur | 2003 | Germany |
| 127 | TG147 | TG147 | Asketis | 1998 | Germany |
| 128 | TG148 | TG148 | Aristos | 1997 | Germany |
| 129 | TG149 | TG149 | Zentos | 1989 | Germany |
| 130 | TG150 | TG150 | Diplomat | 1966 | Germany |
| 131 | TG152 | TG152 | Basalt | 1980 | Germany |
| 132 | TG153 | TG153 | Kormoran | 1973 | Germany |
| 133 | TG154 | TG154 | Aron | 1992 | Germany |
| 134 | TG156 | TG156 | Aszita | 2005 | Germany |
| 135 | TG158 | TG158 | Carimult | 1975 | Germany |
| 136 | TG159 | TG159 | Admiral | 1968 | Germany |
| 137 | TG160 | TG160 | Vuka | 1975 | Germany |
| 138 | TG161 | TG161 | Benno | 1973 | Germany |
| 139 | TG162 | TG162 | Apollo | 1984 | France |
| 140 | TG163 | TG163 | Aquila | 1979 | Italy |
| 141 | TG166 | TG166 | Caribo | 1968 | Germany |
| 142 | TG167 | TG167 | Butaro | 2009 | Germany |
| 143 | TG168 | TG168 | Konsul | 1990 | Germany |
| 144 | TG169 | TG169 | Ares | 1983 | Germany |
| 145 | TG170 | TG170 | Centurk | 2014 | USA |
| 146 | TG171 | - | NS_22_92 | 2007 | Serbien |
| 147 | TG172 | TG172 | Benni_mu | 2015 | USA |
| 148 | TG173 | TG173 | Hope | 1995 | USA |
| 149 | TG174 | TG174 | Vel | 2001 | Germany |
| 150 | TG175 | TG175 | Phoenix | 1981 | AUS:New-South-Wales |
| 151 | TG176 | TG176 | Mironovs | 1970 | Ukraine |
| 152 | TG177 | TG177 | Caphorn | 2001 | UK |
| 153 | TG178 | TG178 | Cordiale | 2005 | England |
| 154 | TG179 | TG179 | Apache | 1998 | Czech Republic |
| 155 | TG181 | TG181 | Isengrai | 1997 | France |
| 156 | TG182 | TG182 | Alixan | 2005 | France |
| 157 | TG183 | TG183 | Boregan | 2008 | France |
| 158 | TG185 | TG185 | Tremie | 1992 | France |
| 159 | TG187 | TG187 | Triple_d | 2016 | Australia |
| 160 | TG188 | TG188 | Cardos | 1998 | Germany |
| 161 | TG189 | TG189 | Soissons | 1988 | France |
| 162 | TG190 | TG190 | BCD_1302 | 2012 | Maldovien |
| 163 | TG191 | TG191 | Arlequin | 2007 | France |
| 164 | TG192 | TG192 | Sonalika | 1978 | Indien |
| 165 | TG193 | TG193 | Camp_Rem | 1980 | France |
| 166 | TG194 | - | Cajeme_7 | 1971 | Mexico |
| 167 | TG195 | - | Avalon | 2016 | UK |
| 168 | TG196 | TG196 | Ivanka | 1999 | Serbien |
| 169 | TG197 | TG197 | Pobeda | 1998 | Serbien |
| 170 | TG198 | TG198 | NS_66_92 | 2015 | Serbien |
| 171 | TG199 | TG199 | Mex_3 | 2003 | Mexico |
| 172 | TG200 | - | Orcas | 2010 | Germany |
| 173 | TG201 | TG201 | Nimbus | 1975 | SE |
| 174 | TG203 | - | Florida | 1985 | USA |
| 175 | TG204 | - | Rumor | 2013 | Germany |
| 176 | TG205 | TG205 | Highbury | 2000 | UK |
| 177 | TG206 | TG206 | Siete_Ce | 2000 | Mexico |
| 178 | TG207 | TG207 | Kontrast | 1990 | Germany |
| 179 | TG208 | TG208 | WW_4180 | 2004 | Germany |
| 180 | TG209 | TG209 | INTRO_61 | 2011 | USA |
| 181 | TG210 | - | NS_46_90 | 2014 | Serbien |
| 182 | TG211 | TG211 | Mex_17_b | 2009 | Mexico |
| 183 | TG212 | TG212 | Lambrieg | 2013 | Chile |
| 184 | TG213 | TG213 | Pegassos | 1994 | Germany |

**Supplementary Table S2** List of cultivars for highest and lowest Pro and H_2_O_2_ accumulation under drought condition

| **Trait** | **Variety** | **Origin** | **Content (µg/g FW)** | **Category** |
| --- | --- | --- | --- | --- |
| Pro | Zobel | Germany | 84.51 | L |
|  | Akteur | Germany | 98.87 | L |
|  | Famulus | Germany | 142.66 | L |
|  | Alixan | France | 198.38 | L |
|  | Tremie | France | 204.75 | L |
|  | KWS_Sant | England | 230.31 | L |
|  | Progress | France | 254.91 | L |
|  | Highbury | UK | 257.95 | L |
|  | Boxer | Germany | 275.99 | L |
|  | Cardos | Germany | 280.11 | L |
|  | Meister | Germany | 2173.39 | H |
|  | Benno | Germany | 2227.02 | H |
|  | Soissons | France | 2283.99 | H |
|  | TJB_990_15 | UK | 2292.80 | H |
|  | Torrild | Germany | 2304.68 | H |
|  | Aquila | Italy | 2304.96 | H |
|  | Topfit | Germany | 2337.09 | H |
|  | NS_22_92 | Serbien | 2361.51 | H |
|  | Hope | USA | 2395.66 | H |
|  | Kurt | Germany | 2420.55 | H |
| H_2_O_2_ | Urban | Germany | 78.09 | L |
|  | Carenius | Germany | 86.28 | L |
|  | Skalmeje | Germany | 89.73 | L |
|  | Lambrieg | Chile | 90.26 | L |
|  | Knirps | Germany | 91.09 | L |
|  | Kontrast | Germany | 91.95 | L |
|  | Kronjuwe | Czech Republic | 97.82 | L |
|  | Oxal | Germany | 99.06 | L |
|  | Ludwig | Germany | 177.76 | H |
|  | Bussard | Germany | 179.36 | H |
|  | Carimult | Germany | 179.48 | H |
|  | Benni_mu | USA | 179.64 | H |
|  | Alixan | France | 181.11 | H |
|  | Aszita | Germany | 195.60 | H |
|  | Tremie | France | 204.05 | H |
|  | Batis | Czech Republic | 207.04 | H |
|  | Akratos | Germany | 207.13 | H |
|  | Elixer | Germany | 216.53 | H |

Note: Abbreviations: L, low proline; H, High proline, Pro=Proline

**Supplementary Table S3** List of alleles and the corresponding cultivars linked with highest and lowest STI for Pro and H_2_O_2_

| **Trait** | **Variety** | **Category** | **STI** | **Alleles** | **Average value of alleles** |
| --- | --- | --- | --- | --- | --- |
| Pro | Akteur | L | 0.66 | C | C= 1.51, T= 52.23 |
|  | Zobel | L | 0.69 | C |  |
|  | Famulus | L | 1.19 | C |  |
|  | Highbury | L | 1.25 | C |  |
|  | Tremie | L | 1.45 | C |  |
|  | Alixan | L | 1.75 | C |  |
|  | Tommi | L | 1.85 | C |  |
|  | Sokrates | L | 1.87 | C |  |
|  | Greif | L | 2.12 | C |  |
|  | Mulan | L | 2.24 | C |  |
|  | Hope | H | 38.36 | T |  |
|  | Vel | H | 40.77 | T |  |
|  | Camp_Rem | H | 49.70 | T |  |
|  | NS_22_92 | H | 52.26 | T |  |
|  | Phoenix | H | 53.38 | T |  |
|  | Benni_mu | H | 53.66 | T |  |
|  | Centurk | H | 77.54 | T |  |
| H_2_O_2_ | Urban | L | 0.73 | GTA | GTA= 0.91, ACG= 2.68 |
|  | Impressi | L | 0.85 | GTA |  |
|  | Lucius | L | 0.86 | GTA |  |
|  | Robigous | L | 0.89 | GTA |  |
|  | Mulan | L | 0.94 | GTG |  |
|  | Bombus | L | 0.95 | GTA |  |
|  | Cardos | L | 1.04 | GTA |  |
|  | WW_4180 | L | 1.05 | GTA |  |
|  | BCD_1302 | H | 2.20 | ACG |  |
|  | Esket | H | 2.31 | GTG |  |
|  | Highbury | H | 2.36 | GCG |  |
|  | INTRO_61 | H | 2.52 | ACG |  |
|  | Alixan | H | 2.69 | ACG |  |
|  | Tremie | H | 3.00 | ACG |  |
|  | Mironovs | H | 3.01 | ACG |  |

Note: Abbreviation: L, low STI; H, High STI

**Supplementary Table S4** Mean soil moisture content (m^3^m^3^) record under control and drought treatment

| Days | Control | Drought |
| --- | --- | --- |
| 1 | 0.191 | 0.190 |
| 2 | 0.191 | 0.185 |
| 3 | 0.192 | 0.160 |
| 4 | 0.190 | 0.130 |
| 5 | 0.189 | 0.109 |
| 6 | 0.193 | 0.080 |
| 7 | 0.191 | 0.067 |
| 8 | 0.191 | 0.054 |
| 9 | 0.190 | 0.054 |
| 10 | 0.190 | 0.053 |

**Supplementary Table S5** Marker, chromosome (Chr), position, P-value, phenotypic variation (PV), allele, favorable (Fav.) allele, T-test value related to Pro and H_2_O_2_ accumulation under control condition

| **Trait** | **Marker** | **Chr** | **Position** | **P-value** | **PV (%)** | **Allele**  **(Major: Minor)** | **Fav. Allele** | **T-test value** |
| --- | --- | --- | --- | --- | --- | --- | --- | --- |
| Pro | AX-158605765 | 1A | 532785236 | 5.17E-04 | 3.70 | C:T | T | 0.04 |
|  | AX-158557326 | 2A | 755978749 | 9.18E-04 | 3.33 | G:A | G | 0.03 |
|  | wsnp_Ra_c10710_17570054 | 3B | 775826294 | 6.44E-04 | 3.47 | G:A | A | 0.03 |
|  | AX-111014946 | 3B | 776359429 | 7.33E-04 | 3.47 | C:T | T | 0.02 |
|  | AX-158598616 | 4A | 739522404 | 6.99E-04 | 3.81 | G:A | G | 0.03 |
|  | AX-89647929 | 4B | 660137099 | 5.25E-04 | 3.58 | A:T | T | <0.01 |
|  | RAC875_c25733_2477 | 5A | 398191033 | 4.22E-04 | 3.70 | A:C | A | 0.02 |
|  | AX-158524974 | 5B | 588516252 | 1.62E-04 | 4.26 | A:C | C | <0.01 |
|  | Kukri_c637_517 | 5B | 711702379 | 5.42E-04 | 3.55 | A:C | C | <0.01 |
|  | wsnp_Ex_c2207_4136036 | 5B | 712600807 | 5.42E-04 | 3.55 | G:T | G | 0.04 |
|  | wsnp_Ku_c16116_24914991 | 5B | 712601710 | 5.42E-04 | 3.55 | C:A | C | <0.01 |
|  | wsnp_RFL_Contig3269_3313084 | 5D | 452214851 | 7.82E-04 | 3.41 | A:G | G | 0.03 |
|  | AX-158552604 | 6B | 662515354 | 6.03E-04 | 3.54 | C:A | A | 0.01 |
| H_2_O_2_ | AX-158602322 | 2A | 34152277 | 1.80E-04 | 8.22 | A:G | G | <0.01 |
|  | AX-109950638 | 2A | 699433856 | 2.06E-04 | 8.06 | G:T | T | <0.01 |
|  | AX-110541191 | 2B | 12009445 | 5.32E-04 | 7.23 | A:C | C | <0.01 |
|  | Excalibur_c25043_618 | 2B | 797329571 | 6.26E-04 | 7.19 | T:C | C | <0.01 |
|  | BobWhite_c11059_169 | 2D | 32053537 | 3.22E-04 | 7.69 | A:C | C | <0.01 |
|  | wsnp_BE488206B_Ta_2_1 | 6D | 62502596 | 5.26E-04 | 7.00 | T:C | T | <0.01 |

Abbreviations: Pro= proline; H_2_O_2_= hydrogen peroxide

**Supplementary Table S6** Haplotypes block, number of markers in haplotype block (NMHB), chromosome (Chr), haplotype block (HB) size, haplotype allele and favorable allele regarding Pro and H_2_O_2_ accumulation under control

| **Traits** | **Haplotype block** | **NMHB** | **Chr** | **HB size (bp)** | **Haplotype alleles** | **Favorable allele** |
| --- | --- | --- | --- | --- | --- | --- |
| Pro | Pro_1A_Hap4 | 3 | 1A | 242151 | AGC: GGT | GTT |
|  | Pro_5A_Hap2 | 3 | 5A | 3884028 | GAC: TGC | TGC |
|  | Pro_7B_Hap2 | 3 | 7B | 595 | AGA: GAG | GAG |
| H_2_O_2_ | HP_1B_Hap3 | 3 | 1B | 24816 | ACG: GTG | ACG |
|  | HP_2B_Hap1 | 11 | 1B | 2193600 | GCGCTGTTTGT: ATATCACCCAT | ATATCACCCAT |
|  | HP_6B_Hap1 | 4 | 6B | 164905 | CTAT: TTGC | TTGC |

Abbreviations: Pro= proline; H_2_O_2_= hydrogen peroxide

**Supplementary Table S7a** Correlation of Pro, H_2_O_2_ and yield-related traits under drought stress condition

|  | **PH** | **GY** | **PBW** | **SDW** | **SN** | **KN** | **TKW** | **DrH_2_O_2_** |
| --- | --- | --- | --- | --- | --- | --- | --- | --- |
| **GY** | 0.11 |  |  |  |  |  |  |  |
| **PBW** | 0.23 | 0.96**** |  |  |  |  |  |  |
| **SDW** | 0.32* | 0.79**** | 0.89**** |  |  |  |  |  |
| **SN** | -0.05 | 0.49*** | 0.48*** | 0.53**** |  |  |  |  |
| **KN** | -0.11 | 0.66**** | 0.55**** | 0.48*** | 0.54**** |  |  |  |
| **TKW** | 0.14 | 0.46*** | 0.52*** | 0.42** | -0.12 | 0.18 |  |  |
| **DrH_2_O_2_** | 0.03 | -0.11 | -0.06 | 0.02 | 0.09 | -0.14 | -0.25 |  |
| **DrProl** | 0.24 | -0.21 | -0.18 | 0.1 | 0.08 | -0.06 | -0.25 | 0.25 |

Note: *p<0.05 (2-tailed); **p<0.01 (2-tailed), and ***p<0.0001 (2-tailed); Abbreviations: GY, grain yield; PBW, plant dry biomass weight; SDW, shoot dry matter weight; SN, spike number; KN, kernels number; TKW, thousand kernel weight (TKW); DrH_2_O_2_, hydrogen peroxide under drought stress; DrProl, proline content under drought stress

**Supplementary Table S7b** Correlation of proline and H_2_O_2_ with yield-related traits under well water (control) condition

|  | **PH** | **GY** | **PBW** | **SDW** | **SN** | **KN** | **TKW** | **Pro_C** |
| --- | --- | --- | --- | --- | --- | --- | --- | --- |
| **PH** |  |  |  |  |  |  |  |  |
| **GY** | 0.36* |  |  |  |  |  |  |  |
| **PBW** | 0.46** | 0.96**** |  |  |  |  |  |  |
| **SDW** | 0.55*** | 0.79**** | 0.93**** |  |  |  |  |  |
| **SN** | 0.07 | 0.58**** | 0.58**** | 0.54*** |  |  |  |  |
| **KN** | 0.1 | 0.90**** | 0.83**** | 0.63**** | 0.67**** |  |  |  |
| **TKW** | 0.63**** | 0.60**** | 0.64**** | 0.62**** | 0.15 | 0.23 |  |  |
| **Pro_Con** | 0.06 | 0.01 | -0.06 | -0.07 | 0.04 | -0.04 | 0.01 |  |
| **H_2_O_2__Con** | 0.13 | 0.09 | -0.08 | -0.05 | -0.04 | -0.11 | 0.02 | 0.15 |

Note: *p<0.05 (2-tailed); **p<0.01 (2-tailed), and ***p<0.0001 (2-tailed); Abbreviations: GY, grain yield; PBW, plant dry biomass weight; SDW, shoot dry matter weight; SN, spike number; KN, kernels number; TKW, thousand kernel weight (TKW); H_2_O_2__Con, hydrogen peroxide under control condition; Pro_Con, proline content under control treatment
