# Supplementary material for: Pinpointing genomic loci for drought-induced proline and hydrogen peroxide accumulation in bread wheat under field conditions
Source: BMC Plant Biol. 2022 Dec 13;22:584. doi: 10.1186/s12870-022-03943-9 (PMC9746221; doi:10.1186/s12870-022-03943-9)
Supplement: Supplementary file 2 — Additional file 2. [file 12870_2022_3943_MOESM2_ESM.docx]

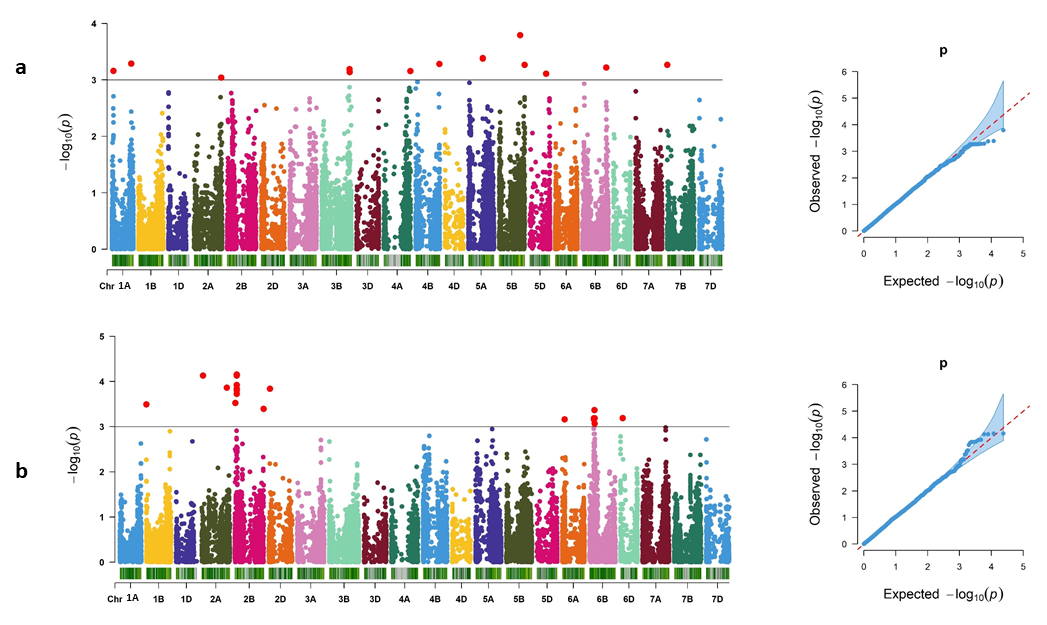


**Supplementary Fig. S1** Manhattan plots of GWAS conducted on Pro and H_2_O_2_ accumulation. (a) Manhattan plot (left) and QQplot (right) of Pro content under control condition, (b) Manhattan plot (left) and QQplot (right) of H_2_O_2_ content under control condition. − log_10_ (P) =3.0 is the significant threshold level for marker-trait association represented by a horizontal grey line on Manhattan plots. Red dots above the horizontal grey line are representing significant markers

**
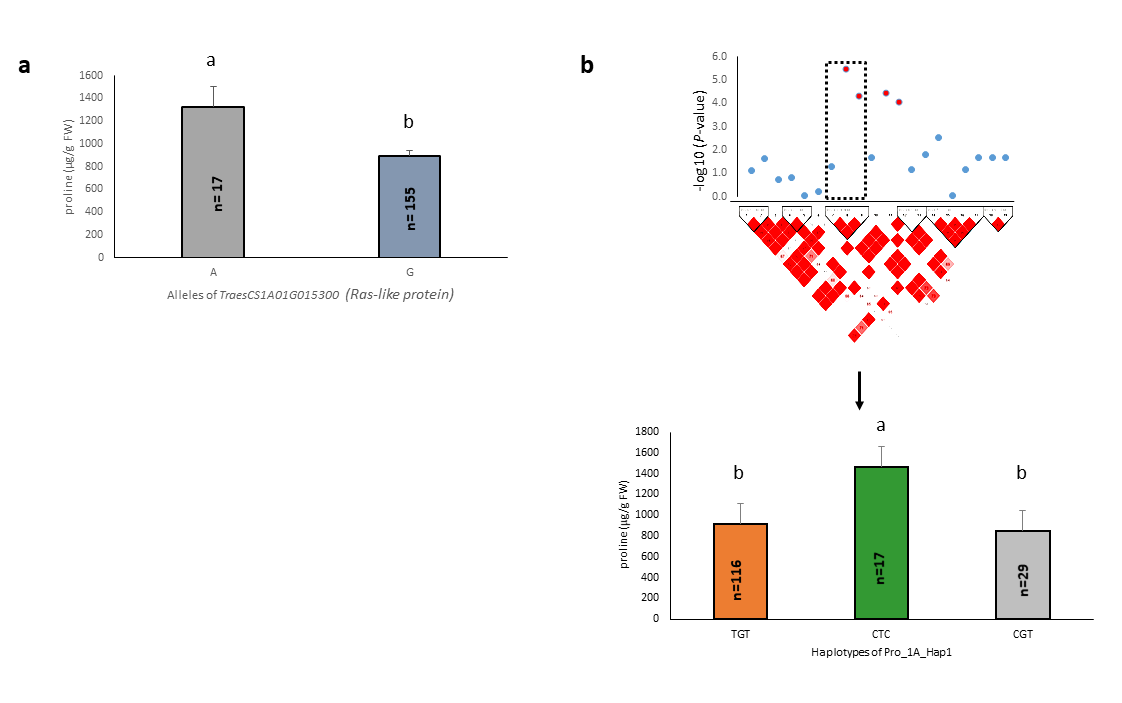
**

**Supplementary Fig. S2** Alleles of candidate gene and haplotype block Pro_1A_Hap1 linked with Pro content under drought. (a) Two allelic forms of the candidate gene *TraesCS1A01G015300* that located on 1A chromosome and harbored significant SNP AX-158569423 in the 4^th^ intron*.* (b) Three haplotypes for Pro_1A_Hap1 haplotype block that were linked with four serine/threonine-protein kinase coding genes. n, number of cultivars possessing the allele or haplotype. Different letters on the bar plots indicating the significant statistical difference.


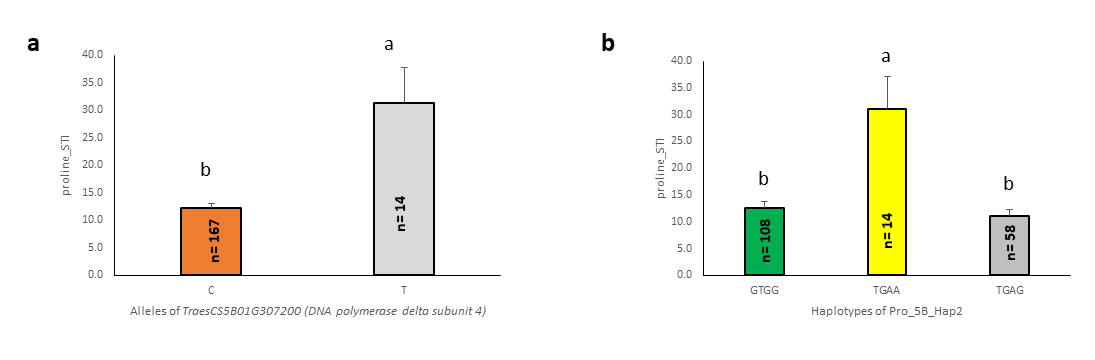


**Supplementary Fig. S3** Alleles of candidate gene and haplotype block Pro_5B_Hap2 linked with STI of Pro. (a) Two allelic forms of the candidate gene *TraesCS5B01G307200* that located on 5B chromosome and harbored significant SNP AX-158525047 in the promoter region. (b) Three haplotypes for Pro_5B_Hap2 haplotype block that was nested with the promoter regions of *TraesCS5B01G321800.* n, number of cultivars possessing the allele or haplotype. Different letters on the bar plots indicating the significant statistical difference.

*
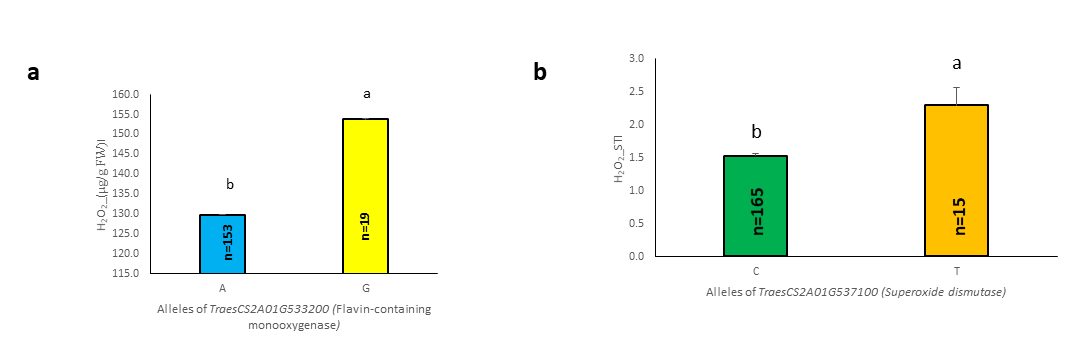
*

**Supplementary Fig. S4** Alleles of two candidate genes, *TraesCS2A01G533200* and *TraesCS2A01G537100* linked with drought and STI of H_2_O_2,_ respectively. (a) Two allelic forms of the candidate gene *TraesCS2A01G533200* that located on 2A chromosome and harbored significant SNP AX-158557366 in the promoter region (697 bp downstream). (b) Two allelic forms of the candidate gene *TraesCS2A01G537100* that located on 2A chromosome and harbored significant SNP AX-158596005 in the first exon. n, number of cultivars possessing the allele. Different letters on the bar plots indicating the significant statistical difference.
